# Natural Compounds for the Treatment of Cutaneous Squamous Cell Carcinoma: A Systematic Review

**DOI:** 10.3390/ijms27125531

**Published:** 2026-06-18

**Authors:** Natalia Forno-Bell, Sara Arciniegas Ruiz, Helena Walker, Seyed Pouya Aghili

**Affiliations:** Department of Biomedical Sciences, Ross University School of Veterinary Medicine, P.O. Box 334, Basseterre KN0202, Saint Kitts and Nevis

**Keywords:** cutaneous squamous cell carcinoma, natural compound, phytochemical, SCC, skin cancer

## Abstract

Cutaneous squamous cell carcinoma (cSCC) is one of the most common non-melanoma skin cancers worldwide. Although surgery and adjuvant therapies are often effective, the treatment of high-risk or advanced lesions remains challenging due to recurrence, resistance, toxicity, and limited long-term control. Natural compounds have, therefore, gained interest as multi-target agents for cancer prevention and treatment. This systematic review aimed to evaluate the antitumoral activity of natural compounds against cSCC. A systematic literature search was conducted following PRISMA 2020 guidelines. Sixty studies met the inclusion criteria and were analyzed using a conservative, mechanism-based classification framework. The included studies evaluated purified compounds, crude extracts, essential oils, formulations, and combination treatments. Despite chemical diversity, antitumoral activity converged on defined biological processes, including apoptosis, non-apoptotic regulated cell death, redox modulation, oncogenic signaling inhibition, cell-cycle arrest, epigenetic regulation, photodynamic ROS generation, and chemopreventive or immune-mediated mechanisms. Mechanistic specificity was higher among purified compounds, while complex extracts showed broader, context-dependent effects. Several agents demonstrated consistent in vitro and in vivo activity, which supports their translational relevance. Natural compounds target shared biological vulnerabilities in cSCC through mechanistically convergent pathways. The framework presented here supports mechanism-guided prioritization and may facilitate the translation of promising compounds into clinically relevant strategies.

## 1. Introduction

Cutaneous squamous cell carcinoma (cSCC) is one of the most common forms of non-melanoma skin cancer and represents a growing public health concern worldwide [1,2]. Although many cases are treated successfully in early stages, a significant proportion of cSCC lesions show aggressive behavior, local recurrence, or metastatic potential. Tumors arising in anatomically sensitive areas or in high-risk patients can be difficult to manage and are associated with increased morbidity. As incidence rates continue to rise globally, there is an increasing need for alternative and complementary therapeutic strategies [2,3].

The primary etiological factor in the development of cSCC is chronic exposure to ultraviolet (UV) radiation, particularly UVB radiation. UV-induced DNA damage, oxidative stress, and sustained inflammatory responses contribute to the malignant transformation of keratinocytes. Additional risk factors include fair skin phototypes, advanced age, immunosuppression, and exposure to chemical carcinogens [1,4,5]. At the molecular level, cSCC is characterized by the dysregulation of multiple signaling pathways involved in cell proliferation, apoptosis, differentiation, and immune evasion. These include alterations in p53 function, aberrant activation of MAPK and PI3K/Akt pathways, and persistent inflammatory signaling, which highlights the complex and multifactorial nature of this disease [1,2].

Surgical extraction is the gold standard of care, followed by radiotherapy, topical agents, or systemic therapies used in selected cases. While these approaches are effective in many patients, they present important limitations. Surgical treatment may be challenging in advanced or cosmetically sensitive lesions, and systemic therapies can be associated with toxicity, high costs, and limited long-term efficacy [2,3]. Moreover, conventional treatments are often directed toward single molecular targets, which may reduce their effectiveness in a tumor type driven by multiple interconnected pathways. These limitations support the exploration of multi-targeted therapeutic approaches [4,6].

Natural compounds derived from plants have attracted increasing interest as potential anticancer agents due to their broad spectrum of biological activity and generally favorable safety profiles. Numerous phytochemicals, including curcumin, resveratrol, epigallocatechin gallate (EGCG), quercetin, and berberine, have shown antitumoral activity in experimental models of skin carcinogenesis [7,8]. These compounds are reported to modulate key processes involved in cSCC development, such as apoptosis, cell-cycle arrest, oxidative stress regulation, inhibition of oncogenic signaling, and suppression of chronic inflammation. Importantly, many natural compounds act on multiple molecular targets simultaneously, which may offer advantages over conventional single-target pharmaceuticals [6,9].

Despite the growing number of studies evaluating natural compounds in cSCC models, the existing evidence remains dispersed and methodologically heterogeneous [10]. Differences in experimental systems, compound classes, and outcome measures limit direct comparison between studies [6,7]. In addition, many reviews address natural compounds in skin cancer broadly, without focusing specifically on cSCC or providing a mechanistic organization of the data. A structured synthesis based on both chemical class and mechanism of action is currently lacking [8,9].

The objective of this systematic review is to comprehensively summarize the anticancer activity of natural compounds against cSCC by analyzing and organizing the available evidence according to chemical structure and dominant molecular mechanisms of action. This integrative and mechanism-based framework aims to support future molecular research and contribute to the rational development of natural compound-based strategies for cSCC prevention and treatment.

## 2. Materials and Methods

### 2.1. Search Protocol and Eligibility Criteria

Scientific literature published prior to February 2026 was collected from Scopus, Web of Science, PubMed, Embase, EBSCO/Medline and Google Scholar databases and systematically evaluated following the PRISMA 2020 checklist [11]. The review protocol was registered in the Open Science Framework (OSF) associated project: https://osf.io/n4jxm/overview (accessed on 2 December 2025). The search terms included “squamous cell carcinoma” OR “SCC” AND “skin” OR “cutaneous” AND “natural compound” OR “phytochemical”.

To be included in this systematic review, a given study had to meet the following initial criteria: original published research, assessing activity of natural compounds on cutaneous squamous cell carcinoma, and reporting relevant outcomes related to antitumoral activity, safety or mechanism. A natural compound was considered to be any bioactive chemical substance derived from plants, fungi, or other natural sources, obtained either as crude extracts or isolated phytochemicals that occur naturally in biological organisms. Reviews, letters or conference abstracts without full dates and studies on any other type of cells, including esophageal squamous cell carcinoma, were excluded. In addition, studies about synthetic drugs, synthetic analogs, or chemically modified derivatives of natural compounds were excluded. No restrictions were imposed regarding the language of publication or the date of publication.

### 2.2. Selection and Data Extraction Procedure

All the search results were imported into EndNote, where duplicates were removed, and then the review process was conducted, where authors N.F.-B. and S.A.R. selected the articles, initially assessing the titles and abstracts of relevant articles to continue examining the full texts of the articles that had been retained from the initial review. Any disagreements that arose during this process were resolved through discussion, which ensured a consensus on the selected studies to avoid biases. Data related to the compounds, their structures, the plants from which they were obtained, cell lines, the type of test to evaluate the antitumoral activities, and the results were extracted from studies by the authors H.W. and S.P.A. in first instance and then rechecked by N.F.-B. and S.A.R.

### 2.3. Data Synthesis

**Figure 1:**
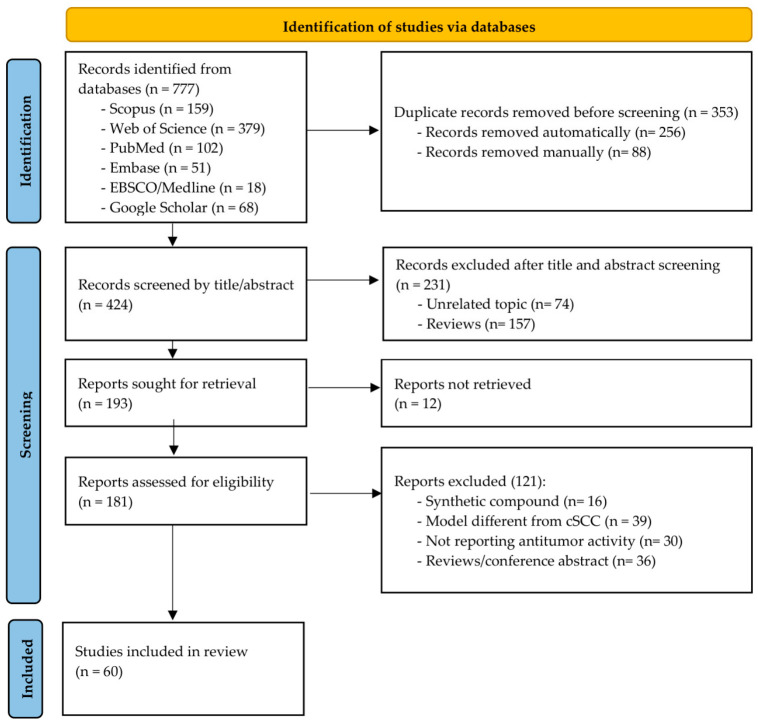
Distribution of natural-product interventions. Identification of studies via database search with 777 articles. Duplicate articles were removed. All articles with titles or abstracts not related to cutaneous squamous cell carcinoma were excluded. After a close review of the full text by two blinded investigators, and after discarding inaccessible articles, 60 articles were retained for a final review.

This work presents a systematic review that addresses data synthesis and analysis. It begins with an overview of the studies, followed by systematic categorization summarized in the flow diagram (Figure 1), following Page et al., 2021 [11]. Following the style used by Ndebia and Kamsu, 2025 [12], a summary table was created to synthesize the included studies, highlighting the experimental models, tests employed, study location, and main findings arranged in descending chronological order (Table 1). In addition, a comprehensive summary table was created to classify natural compounds according to type of preparation and to categorize the compounds by a single dominant mechanism of action reported among the articles (Table 2A–E).

Data extraction focused on variables that were consistently reported across studies, including compound class, preparation type, experimental model, reported IC_50_ values (when available), and dominant mechanism of action. Regarding parameters that are relevant to translational interpretation, such as dosing regimens, routes of administration, treatment duration, and sample size for in vivo studies, Supplementary Table S1 is provided.

Due to the substantial methodological heterogeneity of the included studies, a formal risk-of-bias assessment using standardized tools was not performed. However, common methodological limitations and potential sources of bias were qualitatively identified during data extraction and considered in the interpretation of the findings.

## 3. Results

### 3.1. Overview of Included Studies and Mechanistic Scope

This systematic review included experimental studies evaluating natural compounds and natural-product-derived formulations with antitumoral activity against cutaneous squamous cell carcinoma (cSCC). The studies originated from a broad range of geographic regions, including Asia, Europe, the Middle East, and the Americas, reflecting global research interest in plant-, fungal-, and microorganism-derived anticancer agents. Most investigations were conducted in Asian countries, particularly India, followed by North America and Europe and the Middle East. This geographic distribution parallels the strong tradition of natural-product research and ethnopharmacology in these regions, as presented in Table 1.

Regarding experimental design, the literature was dominated by in vitro studies using established human cSCC cell lines, most commonly A431, SCC-13, and SCL-1. A substantial subset of studies incorporated in vivo validation, primarily through DMBA- or DMBA-/TPA-induced murine skin carcinogenesis models, A431 xenograft models, or UV-induced cSCC models. A smaller but methodologically important group of studies focused on photodynamic therapy (PDT), where light activation was essential for cytotoxicity, as well as immune-dependent chemopreventive models, in which tumor regression occurred without direct cancer cell killing, and in silico docking and molecular dynamics assays (Table 1).

**Figure 2:**
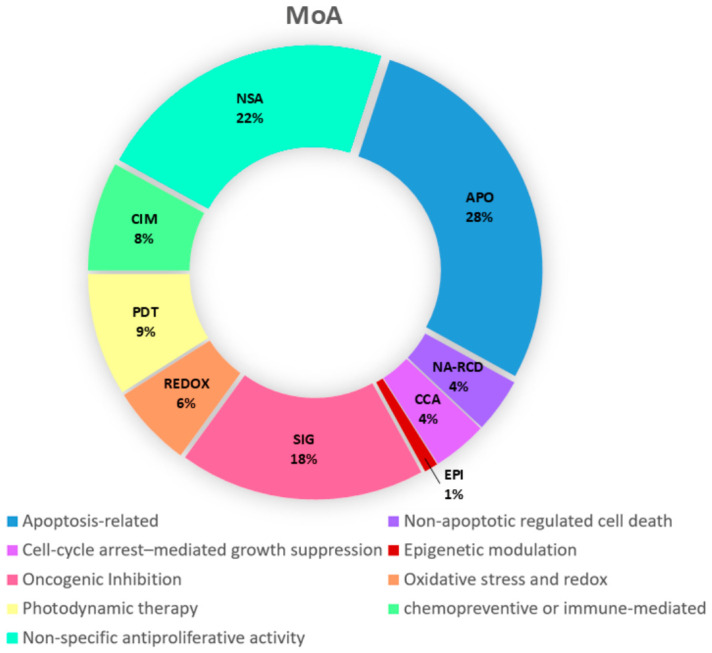
Distribution of natural-product interventions according to mechanism of action (MoA). The pie chart illustrates the relative distribution of compounds across predefined MoA categories. Apoptosis-related mechanisms (APO) represented the largest proportion (26%), followed by non-specific antiproliferative activity (NSA) (22%) and inhibition of oncogenic signaling pathways (SIG) (18%). Mechanisms involving photodynamic therapy (PDT) (9%), oxidative stress and redox regulation (REDOX) (8%), and chemopreventive or immune-mediated mechanisms (CIM) (8%) formed intermediate groups. Less frequently represented mechanisms included non-apoptotic regulated cell death pathways (NA-RCD) (4%), cell-cycle arrest-mediated growth suppression (CCA) (4%), and epigenetic modulation of gene expression (EPI) (1%). Overall, the distribution highlights a strong emphasis on apoptosis-centered and signaling-driven cytotoxic mechanisms, alongside a substantial proportion of compounds with broad antiproliferative effects lacking a dominant mechanistic assignment.

Mechanistically, the reported antitumor effects (Table 1) clustered into well-defined biological themes, allowing classification according to the dominant mechanism of action, as summarized in Figure 2. Most compounds ultimately promoted programmed tumor cell elimination, primarily through apoptosis or alternative regulated cell death pathways, while others exerted their effects through cell-cycle arrest, oncogenic signaling inhibition, oxidative stress modulation, epigenetic reprogramming, photodynamic ROS generation, or chemopreventive and immune-mediated mechanisms. Importantly, the depth of mechanistic validation varied considerably across studies, ranging from cytotoxicity screening assays to detailed pathway dissection, target-engagement analyses, and in vivo functional confirmation.

From a chemical and organizational standpoint, all interventions were systematically classified according to type of preparation, including purified natural compounds (primary and secondary metabolites) (Table 2A), natural extracts (crude plant and fungal extracts and semi-purified fractions) (Table 2B), essential oils (Table 2C), formulated drug-delivery systems (Table 2D), and non-formulated combination treatments (Table 2E). Purified compounds were further categorized based on established chemical classes and subclasses, whereas complex preparations (extracts, fractions, essential oils, and formulations) were classified according to their dominant or most abundant chemical class. Across all preparation types, secondary plant metabolites predominated, with polyphenols (particularly flavonoids) representing the most abundant and frequently investigated chemical group, followed by terpenoids (including mono-, sesqui-, di-, tri-, and tetraterpenoids). Additionally, though less frequently represented, other classes included stilbenes, xanthones, alkaloids, naphthodianthrones, and phenolic acids. Among natural extracts and formulations, polyphenol-dominant preparations were the most common, whereas essential oils were consistently characterized by volatile, terpenoid-rich profiles. This hierarchical classification enabled a structured comparison across chemically defined compounds and multicomponent natural preparations while preserving the dominant phytochemical context underlying their reported antitumoral activity.

For classification purposes, compounds were assigned to a single dominant mechanism of action corresponding to the experimentally validated process that primarily accounted for tumor growth suppression or elimination, even when additional upstream or secondary effects were reported (Figure 2; Table 2A–E).

In addition to these characteristics, despite the absence of a formal risk-of-bias assessment, several recurring methodological limitations were identified across the included studies. Consistent with the experimental distribution described above, the predominant reliance on in vitro models, particularly those based on a limited number of established cSCC cell lines such as A431, restricts the ability to capture tumor heterogeneity. Although a subset of studies incorporated in vivo validation using DMBA-/TPA-induced carcinogenesis models, xenografts, or UV-induced models, these were less frequent and often lacked detailed reporting of methodological aspects such as randomization or blinding. In addition, variability in experimental conditions, including dosing strategies, exposure times, and outcome definitions, limits direct comparability between studies. The frequent focus on selected mechanistic endpoints, together with the predominance of positive findings, could also suggest the potential presence of bias.

### 3.2. Apoptosis-Centered Antitumor Mechanisms

Among all the articles reviewed, apoptosis induction was the mechanism most frequently described for natural products active against cutaneous squamous cell carcinoma. In both in vitro and in vivo models, this mechanism was consistently observed across chemically diverse compounds, including purified primary and secondary metabolites, natural extracts, formulations and non-formulated combination treatments. Across studies, apoptosis-inducing compounds exhibited IC_50_ values primarily within the ~10–50 µM range for purified compounds, with more potent agents reaching values below 10 µM. Extract-based systems generally showed cytotoxic activity between ~20 and 100 µg/mL (Table 1). In vivo studies reporting tumor burden demonstrated reductions typically in the range of ~30–60%, supporting the biological relevance of apoptosis as a dominant antitumoral mechanism in cSCC (Supplementary Table S1).

#### 3.2.1. Purified Natural Compounds

Purified primary metabolites including butyric acid, tetracosane, calcium glucarate, nicotinamide and some secondary metabolites such as cryptolepine, 2′-hydroxycinnamicadehyde, capsaicin and resiniferatoxin have been reported as apoptosis-inducing agents in human cSCC cell lines through kinase inhibition, DNA-damage-driven apoptosis and mitochondrial apoptosis, sometimes accompanied by suppression of tumor-promoting and transformation-associated signaling pathways (MAPK/Akt/NF-κB/AP-1) [34,36,37,51] but reducing mutant p53 expression in in vivo chemical skin carcinogenic DMBA models as well [69]. Notably, 100% of the purified primary metabolites included in this review were associated with apoptosis as their exclusive dominant mechanism of action (Table 2A).

Among secondary metabolites, approximately 75% of simple phenolic compounds demonstrated apoptosis as their primary mechanism of action, whereas this proportion was lower among polyphenols (24%) (Table 2A). Representative examples include flavonoids such as apigenin, fisetin, and licochalcone B, which induce apoptosis in human cSCC cell lines through caspase activation and mitochondrial apoptosis and modulation of Bcl-2 family proteins. These effects were frequently accompanied by cell-cycle arrest, most commonly at the G_2_/M phase, accumulating sub-G_1_ cell populations [33,42,44]. Terpenoid compounds, including ursolic acid, diosgenin, bartogenic acid, glycyrrhizic acid, subamolide B and thymoquinone, are the largest groups exhibiting pro-apoptotic activity. In vitro, these agents induced mitochondrial apoptosis characterized by cytochrome c release, increased Bax/Bcl-2 ratio, and caspase activation, often with higher selectivity toward SCC cells than toward normal keratinocytes [17,38,43,45,60]. In in vivo chemical carcinogenesis models, bartogenic acid reduced tumor incidence in a dose-dependent manner, tumor burden, and histological markers of malignancy [60].

#### 3.2.2. Natural Extracts and Semi-Purified Fractions

Extracts from *Nepenthes miranda*, *Uncaria tomentosa*, *Helichrysum odoratissimum*, and *Punica granatum* induced apoptotic morphology, DNA fragmentation, BAX regulation and caspase activation in the human cSCC cell line A431, often alongside oxidative stress and impaired clonogenic survival [15,34], even when some different solvents—which lead to differences in the extracts—were obtained from the same source [15,31,34]. Nevertheless, enriched fractions from *Helichrysum odoratissimum* exerted stronger apoptotic activity than isolated inert hydrocarbons, which suggests that apoptosis was driven by polyphenolic or terpenoid constituents rather than by non-reactive lipophilic components. Overall, only 24.1% of the natural extracts included in this review were associated with apoptosis as their dominant MoA.

On the side of in vivo model testing, fresh turmeric paste was reported as an apoptosis inducer in a chemical carcinogenesis model, showing tumor regression correlated with caspase-2/3/8/9 induction, p53 activation, and suppressed VEGF/NF-κB signaling [68].

#### 3.2.3. Formulations, Drug-Delivery Systems and Combination Treatments

Formulation strategies frequently enhanced apoptotic outcomes without altering the intrinsic intracellular mechanism. Lycorine-loaded transfersomes enhanced delivery, leading to intracellular accumulation, with increased apoptosis in human cSCC cell lines and reduced xenograft tumor volume in murine models [55].

Non-formulated combination treatments further reinforced apoptosis as a convergent outcome. Thymoquinone combined with pomegranate extract delayed tumor onset and reduced tumor incidence in a two-stage chemical skin carcinogenesis model, with apoptosis selectively induced in carcinogen-exposed skin [65]. Similarly, the combination of thymoquinone and diosgenin produced synergistic apoptosis in human cSCC cells, which was supported by combination index analysis and PI3K/Akt pathway inhibition. The combination induced stronger apoptotic morphology, DNA fragmentation, and tumor suppression than either compound alone [45]. In chemopreventive combinations, such as butyric acid with nicotinamide and calcium glucarate, enhanced intrinsic apoptosis was associated with reduced mutant p53 expression and suppression of tumor development in vivo [69].

#### 3.2.4. Certainty of Evidence for Apoptosis-Centered Antitumor Mechanisms

The evidence for apoptosis is moderate to high, supported by consistent findings across diverse compounds, models, and assay platforms. Apoptotic endpoints were validated using multiple complementary methods and frequently showed dose dependence and tumor selectivity. Importantly, several studies demonstrated concordant in vivo effects, linking apoptosis to reduced tumor burden and delayed progression. Limitations include heterogeneity in designs and a predominance of preclinical models, but overall, apoptosis represents the most robustly supported mechanism.

### 3.3. Non-Apoptotic Regulated Cell Death Pathways

Beyond classical apoptosis, several natural agents evaluated in cSCC models activate alternative regulated cell death (RCD) programs that are distinct from caspase-dependent apoptosis. Within this category, non-apoptotic RCD mechanisms (NA-RCD)—including necroptosis, ferroptosis, and autophagy-dependent cell death—were strongly associated with specific polyphenolic chemical architectures, particularly binaphthyl polyphenols, chalcones, and diarylheptanoids, as well as mixed-chemistry extracts enriched in these scaffolds. Compounds inducing non-apoptotic regulated cell death, including ferroptosis and necroptosis, showed cytotoxic activity comparable to apoptosis-inducing agents, typically within the ~5–30 µM range. This indicates that alternative cell death pathways are engaged at physiologically relevant concentrations and represent viable mechanisms for targeting apoptosis-resistant cSCC cells (Table 1). These pathways were identified in a relatively small proportion of studies, accounting for approximately 4% of all interventions included in this review (Figure 2).

#### 3.3.1. Purified Natural Compounds

Binaphthyl polyphenols, such as gossypol, preferentially induced necroptotic cell death through profound mitochondrial dysfunction rather than through apoptotic signaling. Gossypol caused bioenergetic collapse, ATP depletion, and the loss of mitochondrial respiratory capacity, accompanied by RIP1/RIP3 activation and the absence of caspase engagement, which confirms necroptosis as the dominant mechanism while sparing normal keratinocytes [39].

Chalcone-based polyphenols, such as echinatin, primarily triggered ferroptosis, a caspase-independent RCD pathway driven by iron-dependent lipid peroxidation. Echinatin disrupted cellular redox homeostasis by targeting GSTM3 and PRDX2, promoting mitochondrial ROS accumulation and suppressing ferroptosis-negative regulators (GPX4, xCT, FTH1), ultimately leading to iron-dependent lipid peroxidation and caspase-independent cell death [21].

#### 3.3.2. Natural Extracts and Semi-Purified Fractions

Diarylheptanoids, particularly curcumin, and mixed-chemistry extracts derived from *Curcuma longa* L. predominantly engaged autophagy-dependent cell death [54]. Both isolated curcumin and turmeric extract promoted mTOR-independent autophagic flux and degradation of mutant p53, functioning as the primary cytotoxic mechanism, with apoptosis acting as a secondary or context-dependent outcome.

Overall, these findings that specific polyphenolic scaffolds and extract compositions systematically engage specific NA-RCD pathways, particularly under conditions of mitochondrial failure, redox imbalance, or proteostatic stress, provide effective strategies for eliminating apoptosis-resistant SCC cells.

#### 3.3.3. Certainty of Evidence for Non-Apoptotic Regulated Cell Death Pathways

The evidence for non-apoptotic regulated cell death (NA-RCD) is low to moderate. Although individual pathways such as ferroptosis and necroptosis are supported by highly specific mechanistic assays, including rescue experiments, they are typically described in isolated studies, with limited replication. Ferroptosis shows the strongest support within this category, including in vivo validation. However, the narrow evidence base and limited cross-model validation restrict overall confidence.

### 3.4. Cell-Cycle Arrest—Mediated Growth Suppression

Some natural compounds inhibited cutaneous squamous cell carcinoma growth primarily through cell-cycle arrest, resulting in sustained suppression of proliferation rather than immediate cytotoxicity (purified: ~10–50 µM, extracts: ~20–100 µg/mL) (Table 1). In these studies, disruption of cell-cycle progression was the dominant experimentally demonstrated mechanism, often preceding or occurring independently of apoptotic cell death. Cell-cycle arrest was most frequently observed at the G_2_/M or G_1_/S checkpoints and was supported by molecular modulation of cyclins, cyclin-dependent kinases, and associated regulatory pathways, demonstrated in vitro [20,61]. Evidence supporting this mechanism was found in approximately 4% of all interventions included in this review (Figure 2).

#### 3.4.1. Purified Natural Compounds

Among purified compounds, the flavone chrysin showed some minimal effects on cell-cycle regulators and correspondingly weak antiproliferative activity, which supports cell-cycle blockade as the dominant mechanism underlying the enhanced efficacy of its derivative, compound 69407, which is structurally related to the natural flavone chrysin [61].

#### 3.4.2. Natural Extracts and Semi-Purified Fractions

Fractions derived from *Ottelia alismoides* induced a marked G_2_/M arrest in A431 cells, which is associated with downregulation of cyclin B1 and CDK1, and reduced progression into mitosis. In these experiments, mitochondrial apoptotic markers appeared at later time points, which indicates that cell-cycle blockade preceded and contributed to subsequent cell death. Some compounds like rescinnamine and ottelione that are present in this extract were studied using molecular docking analysis, and they might be responsible for the inhibition of cyclin B, leading to G2/M phase arrest and subsequent apoptotic induction [20].

#### 3.4.3. Certainty of Evidence for Cell-Cycle Arrest—Mediated Growth Suppression

The evidence for cell-cycle arrest is low to moderate, with consistent reports of G_1_/S or G_2_/M blockade supported by molecular and functional assays. In some cases, arrest precedes apoptosis, which suggests a contributory role. However, the evidence is largely restricted to in vitro systems, with limited replication and minimal in vivo confirmation. As a result, its role as an independent antitumor mechanism remains less robust.

### 3.5. Epigenetic Modulation of Gene Expression

Epigenetic modulation was observed almost exclusively among polyhydroxylated flavonoids, particularly catechin-derived structures from green tea, whose chemical features favor interaction with chromatin-associated enzymes. Within this group, epigallocatechin-3-gallate (EGCG) emerged as the primary epigenetically active compound [48]. EGCG directly inhibited DNA methyltransferases (DNMTs) and histone deacetylases (HDACs), which resulted in chromatin relaxation and the reactivation of silenced tumor suppressor genes in skin cancer cells. This epigenetic reprogramming led to cell-cycle arrest via p21 and p16 re-expression and produced chemopreventive growth suppression rather than acute cytotoxicity.

By contrast, structurally related but non- or weakly gallated catechins, including epigallocatechin (EGC) and epicatechin gallate (ECG), exhibited only partial or modest epigenetic effects, without a fully resolved mechanistic link between DNMT/HDAC inhibition and tumor suppressor gene reactivation [48]. Overall, this mechanism of action reflects a structure-dependent capacity of catechol-rich, gallated flavonoids to modulate epigenetic machinery, favoring long-term transcriptional reprogramming over direct tumor cell death.

#### Certainty of Evidence for Epigenetic Modulation of Gene Expression

The evidence for epigenetic modulation is low and largely confined to studies on EGCG. While DNMT/HDAC inhibition and tumor suppressor reactivation are consistently demonstrated at the molecular level, the lack of replication, absence of in vivo validation, and reliance on surrogate endpoints limit confidence. This mechanism remains mechanistically plausible but insufficiently established.

### 3.6. Oncogenic Signaling Pathway Inhibition

The second most common mechanism of action against cSCC demonstrated among these natural compounds was the inhibition of oncogenic signaling pathways that govern proliferation, survival, inflammation, invasion, and tumor promotion. In these investigations, suppression of aberrantly activated pathways—rather than direct induction of cell death—represented the dominant experimentally supported mechanism In both in vitro and in vivo model systems, signaling inhibition was observed across purified compounds, crude extracts, essential oils, standardized formulations, and combination approaches. Compounds targeting oncogenic signaling primarily induced growth inhibition through modulation of pathways such as PI3K/Akt, MAPK, and NF-κB (Table 1). Overall, 18% of the included studies were classified under this mechanism (Figure 2). Of these, approximately 50% corresponded to purified compounds—predominantly polyphenols—while natural extracts accounted for 27.7% and the remaining 22% consisted of essential oils and formulated systems in comparable proportions.

#### 3.6.1. Purified Natural Compounds

Several polyphenolic compounds, particularly flavonoids, but also diarylheptanoids, stilbenes and xanthones, exerted their antitumoral effects primarily through modulation of oncogenic signaling cascades. Compounds such as curcumin, (+)-cyanidan-3-ol, resveratrol, and α-mangostin inhibited key pathways including PI3K/Akt/mTOR, NF-κB, STAT3, Wnt/β-catenin, and MAPK signaling in human cSCC cell lines [30,35,47,56]. This mechanism of action was supported by in vivo validation, reduction of tumor burden, and modulation of histopathological proliferation markers, which underscores its biological relevance.

In the case of flavonoids such as prunetin, tectorigenin, prunetin-4′-O-galactoside, sissotrin, and biochanin-A, the cytotoxicity was demonstrated in vitro, while the EGFR inhibition was supported by in silico docking and molecular dynamics [22]. (+)-Cyanidan-3-ol instead demonstrated inhibition of Akt/mTOR signaling via reactivation of PP2A phosphatase activity, which resulted in reduced SCC cell proliferation, suppression of migration and invasion, and downstream induction of apoptosis. In this context, functional rescue experiments confirmed that pathway inhibition preceded cell death. In addition, for in vivo carcinogenic models, it showed tumor volume and burden reduction [56].

Similarly, α-mangostin exerted strong anti-migratory and anti-invasive effects at non-cytotoxic concentrations in A431 cells, which is associated with reduced expression of matrix metalloproteinases and impaired adhesion signaling. These findings indicate that signaling suppression contributed primarily to reduced metastatic potential rather than direct cytotoxicity [47].

#### 3.6.2. Natural Extracts and Semi-Purified Fractions

Several plant- and fungus-derived extracts inhibited oncogenic signaling in cSCC models, despite their chemical complexity. Methanolic extract of *Ganoderma tsugae* suppressed EGFR activation and downstream PI3K/Akt/mTOR signaling in human cSCC cells, accompanied by reduced VEGF expression and inhibition of angiogenic responses [63].

Extracts from *Azadirachta indica* inhibited tumor development in a two-stage chemical skin carcinogenesis model, primarily through suppression of NF-κB signaling and modulation of STAT-related antiproliferative pathways. Histological analyses indicated tumor degenerative changes rather than dominant apoptotic elimination, consistent with pathway-driven growth suppression [71]. Similarly, for peptide fractions obtained from *Acanthus ebracteatus*, RelA (p65) downregulation and early apoptotic markers were demonstrated without p53 dependence [29].

Polyphenol-rich extracts from *Dalbergia sissoo* also reduced SCC cell viability and proliferation. While strong cytotoxicity was observed, mechanistic support for EGFR inhibition was primarily derived from in silico docking and molecular interaction analyses, with limited functional pathway validation [22].

#### 3.6.3. Essential Oils

Monoterpenoid-dominant essential oils demonstrated antitumor activity through inhibition of oncogenic signaling, particularly in MAPK-driven SCC models.,Essential oils derived from *Mentha aquatica* suppressed aberrant ERK activation in HRAS-mutant keratinocytes, resulting in reduced proliferation, G_2_/M arrest, and attenuation of carcinogenesis in vivo. In these studies, pathway inhibition preceded apoptotic markers, which supports signaling suppression as the primary mechanism [53,58].

#### 3.6.4. Formulations

Formulation strategies frequently enhanced the signaling-modulatory effects of natural compounds without fundamentally altering the underlying mechanism. Liposomal or lipid-based formulations of curcumin increased bioavailability and intracellular accumulation, which resulted in stronger inhibition of STAT3, Akt, and mTOR signaling pathways in vitro and in vivo. In these systems, reduced proliferation and tumor growth were consistently linked to pathway suppression, with apoptosis observed as a downstream or secondary effect [59,62].

#### 3.6.5. Certainty of Evidence for Oncogenic Signaling Pathway Inhibition

The evidence supporting oncogenic signaling inhibition is moderate, with consistent pathway suppression observed across multiple compounds and models. In several cases, temporal and functional validation (including rescue approaches) supports causality. In vivo data further confirm biological relevance. However, heterogeneity in targets, a reliance on surrogate markers, and incomplete validation in some studies limit overall strength.

### 3.7. Oxidative Stress- and Redox-Based Mechanisms

Redox imbalance represents a critical vulnerability of squamous cell carcinoma (SCC) cells, and several natural agents selectively exploit oxidative stress pathways to induce tumor cytotoxicity. Inclusion in this category required direct measurement of ROS modulation and experimental evidence linking oxidative stress to mitochondrial dysfunction, apoptosis, or cytotoxic outcomes. Unlike chemopreventive antioxidants, these agents induced sustained ROS accumulation that exceeded the buffering capacity of SCC cells and directly preceded cell death. Redox-active compounds demonstrated IC_50_ values predominantly within the ~15–60 µM range in vitro, while essential oils and extracts showed activity between ~20 and 100 µg/mL. A clear dose-dependent relationship between ROS accumulation and cytotoxicity was consistently observed, reinforcing oxidative stress as a key driver of tumor cell death (Table 1).

#### 3.7.1. Purified Natural Compounds

Redox-mediated cytotoxicity was consistently observed among redox-active polyphenols and vanilloid phenolics, whose chemical structures favor ROS generation and mitochondrial targeting. Vanilloids, such as [6]-gingerol, induced ROS-dependent mitochondrial dysfunction characterized by the loss of mitochondrial membrane potential, cytochrome c release, caspase-9 and caspase-3 activation, and apoptotic cell death in SCC cells. Importantly, these effects required intact mitochondrial respiration, as respiration-deficient SCC cells were resistant to apoptosis, which confirms a mitochondria-driven redox mechanism [49,51].

Among polyphenols, the xanthone mangiferin induced massive intracellular ROS generation, mitochondrial depolarization, DNA damage, and activation of stress-responsive MAPK signaling, culminating in apoptosis. This response was mechanistically linked to direct interaction with HSP90α, positioning redox imbalance upstream of signaling disruption and cell death [52]. The polymethoxy flavone nobiletin also exhibited redox-modulating activity in SCC models, with antiproliferative efficacy linked to regulation of oxidative stress and mitochondrial involvement, particularly when delivered via optimized topical formulations that enhanced cellular uptake and sustained exposure [57].

#### 3.7.2. Natural Extracts and Semi-Purified Fractions

Among complex preparations, fungal ethyl acetate extracts, particularly from *Aspergillus unguis*, demonstrated potent cytotoxic activity against SCC cells with confirmed ROS involvement. Although these extracts displayed antioxidant activity in chemical scavenging assays, cellular studies revealed pronounced oxidative stress-associated cytotoxicity, underscoring the context-dependent redox-disruptive behavior of secondary metabolite mixtures in tumor cells [28].

#### 3.7.3. Essential Oils

Terpenoid-rich essential oils, including those from *Hedychium spicatum* seeds and *Pamburus missionis*, induced SCC cytotoxicity through ROS-dependent mitochondrial apoptosis. These oils promoted intracellular ROS accumulation, the loss of mitochondrial membrane potential, increased Bax/Bcl-2 ratio, cytochrome c release, caspase activation, and DNA fragmentation. Antioxidant pretreatment abrogated apoptosis, which confirms ROS as the initiating event [25,32]. While isolated essential oil constituents displayed higher intrinsic cytotoxicity than crude oils, the dominant mechanism of the native preparations remained redox-driven mitochondrial dysfunction, which highlights the role of component synergy in oxidative stress induction.

#### 3.7.4. Certainty of Evidence for Oxidative Stress- and Redox-Based Mechanisms

The evidence for redox-mediated mechanisms is moderate, supported by consistent demonstrations of ROS accumulation leading to mitochondrial dysfunction and cell death. Causality is strengthened by antioxidant rescue experiments, and in vivo studies confirm tumor-level effects. However, variability in ROS measurement methods and overlapping with downstream pathways complicate the attribution of redox imbalance as a standalone driver.

### 3.8. Photodynamic Therapy-Related Natural Compounds

A group of natural products exhibited antitumoral activity against cutaneous squamous cell carcinoma specifically under photodynamic conditions, where light activation was required to initiate cytotoxicity. In these studies, photo-induced reactive oxygen species (ROS) generation represented the dominant mechanistic trigger, and cell death occurred only, or predominantly, following irradiation. Photodynamic activity was demonstrated across isolated secondary metabolites, natural extracts and semi-purified fractions in in vitro SCC models. The strict dependence on light distinguishes these compounds mechanistically from conventional redox- or apoptosis-based agents and highlights their potential relevance for localized and controlled treatment strategies in cutaneous squamous cell carcinoma. PDT-related compounds showed minimal activity in the dark (IC_50_ ~70–200 µg/mL) but high cytotoxicity upon light activation (~6–30 µg/mL), which confirms ROS-mediated, light-dependent tumor cell death (Table 1). Approximately 9% of the included studies were classified under this mechanism (Figure 2), predominantly involving polyphenolic purified compounds and polyphenol-rich extracts and fractions, which suggests that chemical features common to polyphenols may favor photodynamic activity, although this association may also reflect a current research focus.

#### 3.8.1. Purified Natural Photosensitizers

Among purified compounds, polyphenol photosensitizers were the most clearly defined photodynamic agents. Curcumin, epigallocatechin gallate, quercetin and hypericin displayed strong light-dependent cytotoxicity in SCC cell models. Mechanistic analyses showed minimal activation of caspase-dependent apoptosis and prominent features of membrane damage and cellular swelling, supporting classification under photodynamically induced non-apoptotic cell death rather than classical apoptosis, where early ROS generation initiated irreversible cell death processes [23,24,46]. Curcumin and hypericin showed the lowest IC_50_ under light activation (Table 1), where epigallocatechin gallate alone showed limited cytotoxicity but significantly restored responsiveness to methyl aminolevulinate (MAL)-based PDT, even despite the cell resistance to this therapy [24].

#### 3.8.2. Natural Extracts and Photosensitizer-Enriched Fractions

Some polyphenol-dominant plant extracts showed pronounced photodynamic properties. Extracts from *Strobilanthes crispa* demonstrated minimal cytotoxicity against human cSCC cells under dark conditions but exhibited a dramatic reduction in IC_50_ values following irradiation at 660 nm [16]. Similarly, fagopyrin F-containing fractions derived from *Fagopyrum tataricum* displayed strong photodynamic cytotoxicity against human cSCC cells. Photo-activated treatment resulted in mitochondrial ROS generation, extensive DNA damage, caspase-3 activation, and apoptotic morphology, while dark toxicity remained minimal [18].

#### 3.8.3. Certainty of Evidence for Photodynamic Therapy-Related Natural Compounds

The evidence for photodynamic mechanisms is low to moderate, characterized by reproducible light-dependent cytotoxicity and ROS generation. The marked contrast between irradiated and dark conditions supports mechanistic specificity and effect size. However, the evidence is limited to in vitro systems, with variable experimental conditions and limited replication, which makes this a promising but still preliminary area.

### 3.9. Chemopreventive and Immune-Mediated Mechanisms

Several agents exerted antitumor effects primarily through chemopreventive or immune-mediated mechanisms rather than through direct cytotoxicity against squamous cell carcinoma (SCC) cells. For the purpose of this review, the inclusion of a compound in this category required in vivo evidence of tumor prevention, delayed malignant progression, microenvironment modulation, or immune dependence rather than acute tumor cell killing. In vivo chemopreventive interventions demonstrated reductions in tumor incidence and progression typically ranging from ~40 to 60%, with some models showing delayed tumor onset and decreased conversion to malignant cSCC. These effects were achieved without overt cytotoxicity, which highlights their relevance for long-term disease control (Supplementary Table S1).

#### 3.9.1. Purified Natural Compounds

Among isolated compounds, chemopreventive activity was mainly associated with stilbenes, phenolic acids, and triterpenoids. Resveratrol acted primarily as an antioxidant and signaling modulator, reducing oxidative DNA damage; inhibiting SCC cell proliferation, migration, and invasion; and suppressing MMP-2 and MMP-9 expression; with limited direct apoptotic activity [30,64]. The structurally related pterostilbene showed stronger chemopreventive efficacy in vivo, reducing tumor incidence and size while enhancing endogenous antioxidant defenses [66].

Ellagic acid, a polyphenol, prevented early carcinogenic changes through antioxidant-mediated protection of genomic integrity [64]. The triterpenoid ursolic acid displayed mixed activity, with moderate direct antiproliferative effects alongside chemopreventive efficacy in inflammation-driven skin carcinogenesis models [38,64]. Overall, isolated compounds in this group favored tumor-suppressive reprogramming rather than dominant cytotoxic mechanisms.

#### 3.9.2. Natural Extracts and Semi Purified Fractions

Polyphenol-dominant extracts, particularly grapeseed extract (*Vitis vinifera*) and proanthocyanidin-rich fractions, demonstrated strong skin cancer-preventive activity. These preparations suppressed tumor initiation and promotion by reducing oxidative stress, limiting aberrant keratinocyte proliferation, and modulating inflammatory pathways, resulting in delayed malignant progression in vivo [64]. Their activity reflects the combined effects of multiple polyphenols on the tumor microenvironment rather than a single dominant intracellular target.

#### 3.9.3. Essential Oils

The terpenoid-rich essential oil camphor white oil (*Cinnamomum camphora*) showed pronounced chemopreventive and immune-mediated effects in vivo. Daily topical application induced regression of premalignant lesions and significantly reduced progression to cutaneous SCC through a T-cell-dependent mechanism, without tumor necrosis or direct cytotoxicity [67]. Whole-oil preparations were more effective than individual constituents, which indicates synergistic immune-modulatory activity intrinsic to the complex essential oil mixture.

#### 3.9.4. Formulations

The polyphenol-based formulation oligonol exhibited robust chemopreventive efficacy in mouse skin carcinogenesis models. Its primary mechanism involved suppression of tumor promotion through inhibition of COX-2 expression, NF-κB activation, and the p38–C/EBP signaling axis, leading to reduced papilloma formation, delayed SCC development, and prolonged survival without overt cytotoxicity [72]. These findings support the classification of oligonol as a signaling-modulatory, chemopreventive agent.

#### 3.9.5. Certainty of Evidence for Chemopreventive and Immune-Mediated Mechanisms

The evidence for chemopreventive and immune-mediated mechanisms is moderate to high, supported by consistent in vivo outcomes, including reduced tumor incidence, delayed progression, and improved survival. Functional validation in some studies (e.g., immune depletion) strengthens causality. However, mechanistic heterogeneity and limited molecular resolution reduce precision despite strong biological relevance.

### 3.10. Compounds Exhibiting Non-Specific Antiproliferative Activity: Antiproliferative Activity Without Dominant Mechanistic Assignment

A subset of natural agents demonstrated reproducible antiproliferative or antitumor effects but lacked sufficient mechanistic resolution within the included studies to assign a dominant intracellular mechanism of action (approximately 22% of the studies reviewed). These studies primarily relied on cell viability assays (MTT/CCK-8/XTT assays), colony formation, tumor growth inhibition, or formulation-enhanced efficacy, without direct initiation of apoptosis, regulated cell death pathways, oncogenic signaling cascades, epigenetic remodeling at the gene level, or immune modulation. To avoid over-interpretation, such agents were conservatively classified as exhibiting non-specific antiproliferative activity (NSA), reflecting limited mechanistic depth rather than the absence of biological effect. Despite limited mechanistic resolution, compounds classified under non-specific antiproliferative activity exhibited comparable potency to mechanistically defined agents. IC_50_ values ranged from ~16 to 60 µM for purified compounds and from ~20 to 150 µg/mL for extracts, in some cases exceeding 400 µg/mL, which indicates that classification reflects insufficient mechanistic characterization rather than reduced biological activity.

Importantly, this classification is study-context-dependent. Several compounds included in this category have well-documented mechanisms of action in other cancer models. However, within the specific experimental frameworks of the studies included in this systematic review, mechanistic interrogation was either not performed or insufficient to support assignment of a dominant pathway. Therefore, NSA classification should not be interpreted as the absence of a mechanism but rather as the absence of an experimentally demonstrated mechanism under the conditions evaluated. This distinction is critical to maintain methodological rigor and avoid extrapolation beyond the evidence base.

#### 3.10.1. Purified Natural Compounds

Several structurally defined natural compounds showed growth-inhibitory effects in squamous cell carcinoma models but were not subjected to sufficient downstream mechanistic validation. Among flavonoids, the green tea catechins (−)-epigallocatechin (EGC) and (−)-epicatechin-gallate (ECG) reduced DNMT and HDAC activity at a global level but did not demonstrate a causal linkage to tumor suppressor gene reactivation or chromatin remodeling comparable to EGCG, which precludes assignment of a dominant epigenetic mechanism of action [48].

Similarly, the flavones tectochrysin and chrysin, as well as labdane-type diterpenoids including 9-hydroxy-hedychenone, hedychilactone-B, -C, and -D, and yunnacoronarin-A, exhibited antiproliferative effects but were evaluated primarily through viability and tumor suppression endpoints, without pathway-specific interrogation [50]. The stilbene resveratrol, when tested alone, showed growth inhibition but lacked mechanistic dissection within the specific experimental context, which supports its classification as non-specifically antiproliferative in this setting [27].

Collectively, these purified compounds exhibited clear antiproliferative activity but insufficient mechanistic resolution to support a dominant mechanism of action assignment.

#### 3.10.2. Natural Extracts and Semi-Purified Fractions

Non-specific antiproliferative activity was the most frequently observed among chemically heterogeneous systems, including crude extracts and semi-purified fractions. Polyphenol-dominant fractions from *Persicaria capitata* and other polygonaceae species demonstrated consistent inhibition of tumor cell growth but did not resolve dominant molecular targets or signaling pathways [13,41].

Likewise, extracts from *Helleborus purpurascens*, Brazilian red propolis (resin from *Dalbergia ecastophyllum*), and *Luisia tenuifolia* demonstrated concentration-dependent growth inhibition in vitro or tumor suppression in vivo, effects attributable to complex polyphenol- and terpenoid-rich mixtures rather than to single validated molecular mechanisms [19,26,70]. Terpenoid-dominant extracts from *Hedychium spicatum* similarly reduced SCC cell viability but were evaluated primarily through phenotypic endpoints without direct interrogation of apoptosis signaling, redox-regulated death pathways, or oncogenic signaling networks [50].

#### 3.10.3. Formulations and Combined Treatments

Formulated delivery systems and combined treatments were also included in this category when enhanced antiproliferative efficacy was reported without mechanistic clarification of intracellular targets. A quercetin nanoemulgel markedly improved cytotoxicity against A431 cells and increased dermal penetration relative to free quercetin; however, its antitumor activity was attributed primarily to improved delivery rather than to elucidation of specific signaling or cell death pathways [14].

Similarly, the combined treatment of ursolic acid and resveratrol, representing a mixed terpenoid–polyphenol system, enhanced growth inhibition and apoptosis relative to individual agents, yet mechanistic analyses did not resolve pathway dominance or synergy at the molecular signaling level, which supports classification as non-specific antiproliferative activity [40].

#### 3.10.4. Certainty of Evidence for Non-Specific Antiproliferative Activity

The certainty of evidence in this category is low, reflecting insufficient mechanistic characterization rather than a lack of activity. Although antiproliferative effects are consistently observed, they are based primarily on phenotypic endpoints, without pathway-specific validation. This group highlights a key limitation of the literature and underscores the need for deeper mechanistic investigation.

## 4. Discussion

This systematic review provides a comprehensive synthesis of natural compounds with antitumoral activity against cutaneous squamous cell carcinoma (cSCC), organized according to chemical class, preparation type, and dominant mechanism of action. Despite the chemical and botanical heterogeneity of the included studies, a clear mechanistic convergence emerged across experimental models, indicating that malignant keratinocytes rely on a limited set of biological pathways for survival and progression. Rather than reflecting non-specific cytotoxicity, the recurrent targeting of mitochondrial integrity, redox homeostasis, oncogenic signaling, and cell-fate regulation suggests the presence of intrinsic vulnerabilities in cSCC cells, consistent with established hallmarks of cancer biology [73,74].

### 4.1. General Mechanistic Interpretation and Biological Convergence

At first glance, the compounds included in this review appear highly diverse, ranging from small, structurally defined phytochemicals to complex extracts, essential oils, formulations, photodynamic agents, and combinatorial treatments. However, when evaluated through the lens of dominant biological effect, their antitumoral activities consistently converged on a restricted number of cellular hubs, namely, mitochondrial dysfunction, oxidative stress dysregulation, suppression of oncogenic survival signaling, and regulation of programmed cell death.

This convergence strongly supports the idea that these pathways represent biological bottlenecks in cSCC rather than incidental pharmacological effects. The repeated engagement of mitochondrial dysfunction across chemically unrelated agents further reinforces the central role of mitochondria as integrators of metabolic stress, redox signaling, and cell death execution in cancer cells [75]. Importantly, these effects were observed across isolated compounds and chemically complex systems, which indicates that distinct chemical entities can exploit the shared vulnerabilities of malignant keratinocytes.

Taken together, the organization of the results by dominant mechanism, rather than by botanical origin alone, proved to be biologically coherent and analytically robust. The observed alignment between chemical architecture, preparation type, and biological response supports the validity of the mechanism-based classification framework applied in this review.

### 4.2. Relationship Between Chemical Structure and Mechanism of Action

A consistent relationship between chemical structure and dominant mechanism of action was particularly evident among purified natural compounds. Lipophilic terpenoids and alkaloids most frequently induced mitochondrial-mediated apoptosis, whereas polyphenolic compounds, such as flavonoids, stilbenes, and xanthones, were more often associated with modulation of oncogenic signaling pathways or redox homeostasis.

Greater mechanistic specificity was observed within defined subclasses. Chalcones and binaphthyl polyphenols preferentially engaged non-apoptotic regulated cell death pathways, including ferroptosis and necroptosis, consistent with their electrophilic and redox-active chemical features [76,77]. By contrast, gallated catechins, particularly epigallocatechin-3-gallate (EGCG), demonstrated a unique ability to interact with chromatin-associated enzymes, leading to epigenetic reprogramming and tumor growth suppression without acute cytotoxicity [48,78].

These observations reinforce the concept that chemical architecture imposes a mechanistic bias and that dominant biological effects cannot be reliably inferred from botanical origin alone. Instead, structure-guided classification more accurately reflects biological activity and translational relevance.

### 4.3. Apoptosis as the Most Frequently Reported Mechanism

Apoptosis emerged as the most frequently reported antitumoral mechanism across the reviewed studies. While apoptosis undoubtedly plays a critical role in cSCC biology, its predominance likely reflects experimental bias rather than exclusive biological relevance. Apoptotic markers are widely available, technically accessible, and routinely incorporated into experimental workflows, whereas alternative regulated cell death pathways require more specialized assays and are often underexplored [79].

Importantly, when mechanistic interrogation extended beyond caspase activation, non-apoptotic cell death programs including necroptosis, ferroptosis, and autophagy-dependent cell death became clearly apparent. This is particularly relevant given that resistance to apoptosis is a well-recognized feature of advanced malignancies, including cSCC [80]. Natural compounds capable of activating alternative death modalities may, therefore, offer valuable strategies for overcoming apoptosis resistance in aggressive or therapy-refractory disease.

### 4.4. Context-Dependent Redox Modulation

Redox regulation emerged as a central but highly context-dependent mechanism. In cytotoxic settings, sustained reactive oxygen species (ROS) accumulation overwhelmed antioxidant defenses, leading to mitochondrial collapse and cell death. By contrast, within chemopreventive and immune-mediated models, redox modulation primarily reduced oxidative DNA damage, suppressed chronic inflammation, and delayed malignant transformation without inducing tumor cell necrosis.

This dual behavior aligns with the established two-face model of ROS in cancer biology, where moderate ROS signaling supports proliferation and survival, whereas excessive or prolonged oxidative stress triggers cell death [81,82]. The clear contextual separation between cytotoxic redox disruption and chemopreventive antioxidant activity justifies their classification as distinct mechanistic categories within the proposed framework.

### 4.5. Influence of Preparation Type on Mechanistic Resolution

Mechanistic resolution closely followed preparation type across the evidence base. Purified compounds allowed for the precise attribution of dominant intracellular mechanisms, whereas chemically complex systems, such as crude extracts, fractions, and essential oils, frequently exerted cumulative or network-level effects. Polyphenol-rich extracts commonly modulated inflammatory signaling or tumor-promotion pathways, while terpenoid-dominant essential oils preferentially induced mitochondrial dysfunction and oxidative stress.

When mechanistic investigation was limited to phenotypic endpoints such as cell viability or tumor burden, conservative classification as non-specific antiproliferative activity was applied. This approach reflects the complexity of botanical mixtures rather than weak biological activity and aligns with best practices in natural-product pharmacology [83,84]. Importantly, this classification reflects limitations in mechanistic resolution within the included studies rather than the absence of an underlying molecular mechanism. Several compounds grouped under non-specific antiproliferative activity have well-established mechanisms of action in other cancer contexts. However, in the studies included in this review, mechanistic interrogation was either not performed or insufficient to support assignment of a dominant intracellular pathway. This conservative approach was adopted to avoid extrapolation beyond the primary data and to maintain consistency in mechanism-based classification across heterogeneous experimental designs.

Extraction solvent strongly influenced phytochemical composition and, consequently, the dominant mechanisms of action observed for natural extracts. However, in some botanical systems, extracts obtained with solvents of different polarity converged mechanistically when a shared triggering condition defined the biological outcome. This was evident for *Strobilanthes crispa*, where extracts of varying polarity consistently produced photodynamic cytotoxicity driven by light-induced ROS generation, which indicates that irradiation, rather than solvent choice, determined the dominant mechanism [16].

By contrast, other botanical sources showed clear solvent-dependent divergence reflecting shifts in dominant chemical drivers. Ethanolic extracts of *Helichrysum odoratissimum* enriched in polyphenols induced apoptosis, whereas non-polar fractions and isolated alkanes were weakly active, which demonstrates that chemical composition, rather than botanical origin, dictated biological effects [34]. A similar pattern was observed for *Nepenthes miranda*, where methanol and ethanol extracts induced apoptosis, DNA damage, and clonogenic suppression, while the acetone extract showed antiproliferative activity with limited mechanistic resolution, which justifies separate mechanism of action assignments despite shared growth inhibition [15]. Divergence was even more pronounced in *Hedychium spicatum*, where chloroform extracts, isolated labdane-type diterpenoids, and the essential oil exhibited distinct cytotoxic mechanisms driven by solvent-defined chemical composition [25,50].

Overall, these findings emphasize the importance of solvent selection and chemical characterization when interpreting extract-based studies and support conservative, mechanism-based classification rather than reliance on botanical source alone.

### 4.6. Photodynamic Therapy as a Distinct Mechanistic Modality

PDT-related natural compounds formed a clearly distinct mechanistic category. In these systems, cytotoxicity was strictly dependent on light-activated ROS generation, with minimal or absent dark toxicity. This strict dependency provides temporal and spatial control over tumor damage and is particularly well suited for cSCC, where lesions are localized and accessible [85,86,87].

Mechanistically, PDT integrates photo-induced redox stress with regulated cell death signaling, which may vary depending on photosensitizer localization and irradiation parameters while remaining functionally distinct from non-photoactivated redox therapies [88]. These features justify the independent classification of PDT within the mechanism of action framework.

### 4.7. Chemoprevention and Immune-Mediated Effects

Chemopreventive and immune-mediated mechanisms were the most prominent in in vivo skin carcinogenesis models. Rather than inducing overt cytotoxicity, several natural agents suppressed malignant progression through modulation of inflammation, immune surveillance, and the tumor microenvironment.

This is highly relevant to cSCC pathogenesis, which frequently arises in chronically inflamed, UV-damaged, or immunocompromised skin. These findings align with established concepts of cancer immunoediting and inflammation-driven carcinogenesis [89,90,91].

### 4.8. Translational Implications and Limitations

The mechanistic patterns identified in this review suggest that the successful translation of natural compounds into clinical strategies may depend on matching mechanism to disease context [92,93]. Apoptosis- and redox-based cytotoxic agents may be more appropriate for established tumors, whereas epigenetic, chemopreventive, and immune-mediated agents appear better suited for early lesions, field cancerization, or long-term prevention. Compounds capable of inducing non-apoptotic regulated cell death may offer valuable options for overcoming resistance in advanced disease [93].

Importantly, several features of natural compounds support their translational potential. Many agents identified in this review demonstrated consistent antitumoral activity across both in vitro and in vivo models, including measurable reductions in tumor burden, incidence, and progression. Representative examples include polyphenols such as apigenin and mangiferin, which showed reproducible cytotoxic and tumor-suppressive effects across multiple SCC models; terpenoid compounds such as ursolic acid and thymoquinone, which demonstrated both in vitro antiproliferative activity and in vivo tumor reduction; and combination or extract-based systems such as thymoquinone with pomegranate extract and polyphenol-rich grape seed preparations, which reduced tumor incidence and progression in chemical carcinogenesis models.

In addition, their ability to simultaneously modulate multiple oncogenic pathways aligns with the multifactorial biology of cSCC and may reduce the likelihood of resistance compared with single-target therapies. This multi-target activity is well illustrated by compounds such as curcumin, resveratrol, and α-mangostin, which concurrently regulate PI3K/Akt, NF-κB, MAPK, and oxidative stress pathways, as well as by echinatin and gossypol, which engage alternative regulated cell death pathways, including ferroptosis and necroptosis.

Several limitations should be acknowledged. The evidence base is dominated by preclinical models, with limited clinical validation. Mechanistic depth varies substantially across studies, and reporting bias toward apoptosis likely underestimates the contribution of alternative death pathways. In addition, heterogeneity in experimental design, dosing, and outcome measures limits quantitative comparison.

Beyond these methodological constraints, several translational barriers further limit clinical application. Many natural compounds (particularly polyphenols such as flavonoids and stilbenes) exhibit poor bioavailability due to low solubility, rapid metabolic clearance, and limited tissue penetration, which results in discrepancies between potent in vitro activity and in vivo efficacy. While formulation strategies (e.g., nanoemulsions, liposomal systems, and topical delivery platforms) have partially addressed these limitations, their implementation remains inconsistent and insufficiently standardized across studies.

For extract-based systems, the lack of standardization represents an additional challenge. Variability in plant source, harvesting conditions, extraction solvents, and preparation methods can lead to significant batch-to-batch differences in phytochemical composition and biological activity. This variability complicates reproducibility, limits cross-study comparability and represents a major obstacle for both mechanistic interpretation and clinical development. In particular, the predominant reliance on single cell line in vitro models, often without validation in more complex systems, may lead to an overestimation of antitumoral activity. Although in vivo studies provide more physiologically relevant insights, they frequently lack detailed reporting of methodological aspects that are critical for internal validity. In addition, the emphasis on selected mechanistic pathways—consistent with the classification approach adopted in this review—may reflect incomplete mechanistic characterization or selective reporting.

Furthermore, current regulatory frameworks are primarily designed for single-entity pharmaceuticals and are not well suited to complex, multi-component botanical mixtures. A practical way forward is to prioritize either (1) chemically characterized lead compounds derived from extracts or (2) simplified, standardized mixtures with well-defined composition, both of which are more compatible with existing regulatory pathways and clinical trial design.

Taken together, these limitations highlight the need for more structured and translationally aligned research strategies. Future work should integrate three key elements: (1) mechanism-guided prioritization of compounds targeting validated cSCC vulnerabilities (e.g., mitochondrial function, redox balance, and oncogenic signaling), (2) early incorporation of pharmacokinetic optimization and delivery strategies, and (3) use of clinically relevant models and quantitative endpoints to enable cross-study comparability.

By aligning compound selection, formulation design, and mechanistic validation within a unified framework, it should be possible to bridge the gap between experimental efficacy and clinical application, ultimately enabling the development of safe, reproducible, and effective natural compound-based therapies for cSCC.

The lack of a formal risk-of-bias assessment represents an additional limitation of this systematic review, and the findings should, therefore, be interpreted with caution. Publication bias toward positive outcomes cannot be excluded, particularly in the context of preclinical research on natural compounds. Taken together, these limitations underscore the need for more standardized, rigorously designed, and transparently reported studies to improve the reliability and translational relevance of the available evidence.

### 4.9. Overall Conclusions

Collectively, the evidence indicates that chemical structure, preparation type, and biological context jointly determine the dominant antitumoral mechanism of natural products in cSCC. Increasing chemical definition correlates with greater mechanistic specificity, whereas increasing complexity promotes network-level modulation. The conservative, mechanism-based framework applied in this review captures these relationships in a biologically coherent manner consistent with molecularly informed cancer models [92] and provides a solid foundation for organizing future experimental studies and guiding the rational translational development of natural compounds for cutaneous squamous cell carcinoma.

## Figures and Tables

**Table 1:** Overview of the antitumoral activity of natural compounds against cSCC models.

Natural Compounds/Extract	Experimental Model	Types of Tests	Antitumoral Activities	Reference (Country)
**In vitro studies**				
*Persicaria capitata* fractions	In vitro (A431)	CVA; ROS	Cell viability reduced; IC_50_ = 475.2 µg/mL (semi-purified fraction); crude extract showed weak cytotoxicity.	[13] (India)
Quercetin	In vitro (A431)	CVA; WHA	Enhanced cytotoxicity versus free quercetin; IC_50_ = 123.3 µM with 66.52% (lowest IC_50_ and highest inhibition); wound-closure migration suppressed.	[14] (India)
*Nepenthes miranda* extracts	In vitro (A431)	CVA; CIAG; Apop; ROS; M; DNA dmg	Cytotoxicity (IC_50_ = 90.61 μg/mL) with apoptosis and DNA damage; clonogenic growth and migration suppressed.	[15] (Taiwan)
*Strobilanthes crispa* extracts	In vitro (A431)	CVA; PDT	Light-activated IC_50_ = 6.1–9.4 µg/mL (660 nm); IC_50_ > 200 µg/mL in dark; photodynamic cytotoxicity confirmed.	[16] (Malaysia)
Glycyrrhizic acid	In vitro (MCC13)	CVA; Apop; CC; MEA	Mitochondrial apoptosis induced; cell-cycle arrest at G_2_/M; selective cytotoxicity; VEGFR-2 expression reduced; IC_50_ = NR.	[17] (Egypt)
Fagopyrin F–containing fraction	In vitro (A431)	CVA; PDT; Apop; ROS; DNA dmg; MEA	IC_50_ = 29.1 µg/mL (light) vs. 69.5 µg/mL (dark); mitochondrial ROS and apoptosis induced.	[18] (India)
*Luisia tenuifolia* extracts	In vitro (A431)	CVA	IC_50_ = 24.3 µg/mL (ethanol extract); IC_50_ ≈ 78.6 µg/mL (ethyl acetate extract).	[19] (India)
*Ottelia alismoides* fraction	In vitro (A431)	CVA; CC; Apop; PE; MitF; MEA	IC_50_ ≈ 10 µg/mL; strong apoptosis at 48 h; G_2_/M arrest; mitochondrial depolarization observed.	[20] (India)
Gossypol	In vitro (SCL-1)	CVA; MitF; PE; NA-RCD	Selective necroptotic cell death; viability reduced at 2–5 µM; RIP1/RIP3-dependent mechanism; minimal effect on normal keratinocytes; IC_50_ = NR.	[21] (Germany)
*Dalbergia sissoo* extracts	In vitro (A431)	CVA; MEA	Cytotoxicity; IC_50_ ≈ 15.37 µg/mL (methanolic extract); in silico EGFR binding supported.	[22] (India)
Quercetin; curcumin; epigallocatechin gallate	In vitro (MET1)	CVA; PTD; ROS	Curcumin IC_50_ = 7.2–26 µM (time dependent, light-activated); quercetin and EGCG IC_50_ > 100 µM and are inactive under PDT.	[23] (Portugal)
Epigallocatechin gallate	In vitro (HSC-1)	CVA; Apop; ROS; PDT; CIAG; WHA; MEA	Restored MAL-PDT efficacy; complete elimination of parental SCC cells; reduced survival of PDT-resistant cells; IC_50_ = NR.	[24] (Switzerland)
*Hedychium spicatum* seed oil	In vitro (A431)	CVA	Cytotoxicity 24–60% (50–200 µg/mL); IC_50_ ≈ 71.2 µg/mL.	[25] (India)
*Helleborus purpurascens* extract	In vitro (A431)	CVA; GE	Moderate growth inhibition; antiproliferative effect without strong cytotoxicity; IC_50_ = NR.	[26] (Romania)
Resveratrol	In vitro (A431)	CVA	Enhanced cytotoxicity with improved epidermal–dermal drug deposition IC_50_ = 22.1 µM versus 52.9 µM for the conventional formulation.	[27] (India)
Fungal isolates	In vitro (A431)	CVA; Apop; CC; MitF; ROS	Viability reduced (IC_50_ (μg/mL): 5.94 ± 0.077 MTT; 6.09 ± 0.051 resazurin); G_1_ arrest; ROS-dependent mitochondrial apoptosis induced.	[28] (India)
*Acanthus ebracteatus*	In vitro (A431)	CVA; Apop; PE	Selective cytotoxicity; IC_50_ ≈ 426 ng/mL; apoptosis induced.	[29] (Thailand)
Resveratrol	In vitro (HSC-5)	CVA; WHA; PE; GE	Proliferation inhibited; migration and invasion suppressed; MMP-2/9 expression reduced; IC_50_ = NR.	[30] (China)
*Uncaria tomentosa* extract	In vitro (A431)	CVA; CC; ROS; PE; DNA dmg	Dose- and time-dependent cytotoxicity (IC_50_ between 1.5 and 2.0 mg/mL at 48 h of treatment); DNA damage induced; apoptosis preferentially in SCC.	[31] (Italy)
*Pamburus missionis* oil	In vitro (A431)	CVA; MitF; ROS; CFA; CC; Apop; GE; PE	IC_50_ ≈ 100 µg/mL; ROS-dependent apoptosis; colony formation suppressed.	[32] (India)
Licochalcone B	In vitro (A431)	CVA; CC; CIAG; Apop; MitF; PE; MEA	IC_50_ = 19.1 µM; pan-caspase activation; colony-forming capacity suppressed.	[33] (Korea)
*Helichrysum odoratissimum* extract	In vitro (A431)	CVA; MEA; ROS	IC_50_ ≈ 15.5 µg/mL; apoptosis induced; crude extract more potent than isolated fractions.	[34] (South Africa)
Curcumin	In vitro (A431)	CVA; Apop; PE	Growth inhibition 44.9–53.4%; STAT3 protein suppressed; apoptosis induced; nanocomplex uptake rapid; IC_50_ = NR.	[35] (India)
Cryptolepine	In vitro (A431, SCC-13)	CVA; CFA; DNA dmg; CC; MitF; Apop; MEA; GE; PE	IC_50_ ≈ 41–58 µM (24–72 h); DNA damage-driven apoptosis; selectivity preserved.	[36] (USA)
2′-hydroxycinnamicadehyde (2′-HCA)	In vitro (A431)	CVA; Apop; CIAG; PE; TumG; TumP	Growth inhibition (IC_50_ ≈ 3 μM); apoptosis increased dose-dependently (up to ~30%); intrinsic mitochondrial pathway activated.	[37] (Korea)
Ursolic acid	In vitro (SCL-1)	CVA; Apop; MEA	IC_50_ = 21.3 µM; apoptosis and G_0_/G_1_ arrest enhanced.	[38] (China)
Turmeric extract and Curcumin	In vitro (A431)	CVA; Apop; Autoph; PE; MEA	Cytotoxicity at 10–20 µg/mL; autophagy-dependent cell death with apoptosis restoration, IC_50_ = NR.	[39] (Thailand)
Ursolic acid + resveratrol	In vitro (Ca3/7)	CVA; Apop; MEA; PE	Viability reduced (IC_50_ of 19.66 ± 3.69 µmol/L); synergistic cytotoxicity; enhanced antiproliferative activity.	[40] (USA)
Polygonaceae extracts	In vitro (A431)	CVA	Proliferation inhibited; strongest activity with extracts, IC_50_ = NR.	[41] (Hungary)
Fisetin	In vitro (A431, SCC-13)	CVA; CC; MitF; Apop; GE; PE	Selective cytotoxicity (dose- and time-dependent); G_2_/M arrest; mitochondrial apoptosis induced, IC_50_ = NR.	[42] (USA)
Subamolide B	In vitro (SCC12)	CVA: Surv; Apop; MitF; GE; CC; MEA	IC_50_ = 9.1 µM (SCC12) and IC_50_ = 13.30 µM (A431); mitochondrial apoptosis; long-term clonogenic survival suppressed.	[43] (Taiwan)
Apigenin	In vitro (A431)	CVA; Apop; CC; ROS; PE; GE; M	Selective growth inhibition; IC_50_ ≈ 15.2 µM; G_2_/M arrest; increased subG_1_ apoptotic fraction.	[44] (Taiwan)
Thymoquinone + Diosgenin	In vitro (A431)	CVA; CC; Apop; PE	Synergistic cytotoxicity A431 (TQ + DG IC_50_ ≈ 10 µg/mL); enhanced apoptosis versus single agents; tumor growth significantly suppressed.	[45] (India)
Hypericin	In vitro (HSCC)	CVA; ROS; PDT; Apop	Photodynamic cytotoxicity at ≥3 µM; ROS burst; caspase-independent necrotic death; IC_50_ = NR.	[46] (South Africa)
α-mangostin	In vitro (A431)	WHA; CVA Adh; GE; M/I	Migration reduced to 6% of control; invasion reduced to 4% at non-cytotoxic doses; IC_50_ = NR.	[47] (Australia)
−(-)Epigallocatechin-3-gallate	In vitro (A431, SCC-13)	PE; GE	Reactivation of p16 and p21 expression; DNMT and HDAC activity reduced; growth suppression without cytotoxicity; IC_50_ = NR.	[48] (USA)
[6]-Gingerol	In vitro (A431)	CVA; CC; ROS; MitF; Apop; GE PE	IC_50_ ≈ 300 µM; apoptosis increased (16–30%); ROS-dependent mitochondrial dysfunction.	[49] (India)
Labdane-type diterpenes from *Hedychium spicatum*	In vitro (A431)	CVA	IC_50_ = 16.0–37.5 µg/mL; strong in vitro cytotoxicity.	[50] (India)
Vanillyl moiety	In vitro (ρ^0^ cells COLO 16, SRB-12)	Apop; MitF; ROS; CC	Apoptosis induced in respiration-competent SCC cells (~60% at 10 µM resiniferatoxin); no apoptosis in ρ^0^ cells; proliferation suppressed via G_1_ arrest in respiration-deficient cells; IC_50_ = NR.	[51] (USA)
**Combined in vivo + in vitro studies**				
Mangiferin	In vitro (A431, COLO-16, SCL-2); in vivo (XG)	CVA; Prolif; CC; Apop; M/I; CIAG; ROS; MitF; DNA dmg; PE; MEA; Histo; Tox	Inhibition of multiple SCC cell lines (IC_50_ (µM) = 42.09 (A431); 58.63 (COLO-16); 50.76 (SCL-2)); tumor volume and weight reduced; massive ROS accumulation; apoptosis and DNA damage induced.	[52] (China)
*Mentha aquatica* L. cv. Lime oil	In vitro (PDV-HRAS Q61L); in vivo (Two-stage CSC, DMBA/TPA)	CVA; CIAG; Apop; CC; PE; ICD; TIC-Prolif	Proliferation suppressed; G_2_/M arrest and caspase-3-dependent apoptosis induced; papilloma incidence and burden reduced; IC_50_ = NR.	[53] (Taiwan)
Echinatin	In vitro (A431, SCL-1); in vivo (XG)	CVA; CIAG; MitF; ROS; PE; M; CFA Ferropt	Proliferation and migration inhibited (dose-dependent); ferroptosis-dependent death; xenograft and UV-induced tumor volume reduced; no systemic toxicity; IC_50_ = NR.	[54] (China)
Lycorine	In vitro (SCL-1); in vivo (XG)	CVA; Apop; Histo	Cytotoxicity (dose-dependent); apoptosis; tumor volume significantly reduced; no systemic toxicity; IC_50_ = NR.	[55] (China)
(+)-Cyanidan-3-ol	In vitro (A431); in vivo (Two-stage CSC, DMBA/TPA)	CVA; CC; Apop; M/I	Selective SCC growth inhibition (time- and dose-dependent); cell-cycle arrest and apoptosis; tumor volume and burden reduced; IC_50_ = NR.	[56] (India)
Nobiletin	In vitro (A431); in vivo (CSC, DMBA)	CVA; ROS	Cell viability reduced (IC_50_ = 16.7 ± 0.9 µg/mL, 48 h); tumor regression and epidermal normalization; Ki-67 expression markedly reduced; antioxidant enzyme levels restored.	[57] (Egypt)
*Mentha aquatica* oil	In vitro (PDV-HRAS^Q61L); in vivo (Two-stage CSC, DMBA/TPA)	CVA; Apop; CC; I; WHA; CFA	Viability reduced; clonogenic growth suppressed (>80% apoptosis at high dose); papilloma incidence and multiplicity reduced; Ki-67-positive keratinocytes decreased; IC_50_ = NR.	[58] (Taiwan)
Curcumin	In vitro (JB6); in vivo (UVB-SC); ex vivo (human IHC)	CVA; CC; PE; Histo; CFA	UVB-induced epidermal thickening reduced; Ki-67-positive cells decreased; FGFR2-mTOR signaling suppressed; IC_50_ = NR.	[59] (USA)
Bartogenic acid	In vitro (SCC-13); in vivo (Two-stage CSC, DMBA/Croton oil)	CVA; Histo; ROS	Incidence reduced; antioxidant enzymes increased; selective cytotoxicity; IC_50_ = NR.	[60] (AEU)
Chrysin	In vitro (A431); in vivo (XG)	CVA; CIAG; CC; TumG	Viability and tumor growth suppressed; G_1_/S arrest; CDK2/4 activity inhibited; IC_50_ = NR.	[61] (USA)
Curcumin	In vitro (SRB12-p9); in vivo (XG)	PE; TumG	Tumor volume reduced; proliferation marker Ki-67 decreased; no systemic toxicity; IC_50_ = NR.	[62] (USA)
*Ganoderma tsugae* extract	In vitro (A431); in vivo (XG)	CVA; PE; I; Histo	Viability reduced; sensitization to paclitaxel; tumor growth suppressed in xenograft model; VEGF expression reduced; IC_50_ = NR.	[63] (Taiwan)
Proanthocyanidin extracts (GSE; RES; URA; ELA)	In vitro (Ca3/7); in vivo (Two-stage CSC, DMBA/TPA)	CVA; Apop; ROS; DNA dmg; CIAG; PE; TumG; TumP	Papilloma multiplicity reduced; tumor promotion suppressed; antioxidant-mediated chemopreventive effects predominant; IC_50_ (μM) = 34.7 GSE, 5.2 RES, 11.5 URA, 51.1ELA.	[64] (USA)
**In vivo studies**				
Thymoquinone + pomegranate extract	In vivo (Two-stage CSC, DMBA/TPA)	Apop; ROS; Histo	Tumor onset delayed (weeks 7 → 13); tumor incidence reduced (100% → 40%); tumor volume and progression significantly reduced; apoptosis 16.8%.	[65] (Saudi Arabia)
Pterostilbene	In vivo (Two-stage CSC, DMBA/TPA)	Histo	Tumor incidence and size reduced; antioxidant enzymes increased; apoptotic markers elevated.	[66] (Malaysia)
Camphor white oil	In vivo (Two-stage CSC, DMBA/TPA)	TB/Inc/MalConv; ImmDep,; CVA RNA-seq	Regression of premalignant lesions; ~50% reduction in SCC conversion; immune-dependent tumor control.	[67] (USA)
Fresh turmeric paste	In vivo (CSC, DMBA)	Histo; Prolif; Apop; GE ImmDep	Tumor volume reduced (0.254 → 0.172 cm^3^); apoptotic index increased (2.7 → 32); VEGF suppressed.	[68] (India)
Butyric acid, Nicotinamide, Calcium glucarate	In vivo (CSC, DMBA)	GE; MitF; PE; Apop	Intrinsic apoptosis enhanced; mutant p53 suppressed; chemopreventive efficacy superior to single agents.	[69] (India)
Brazilian red propolis extract	In vivo (CSC, DMBA)	Histo	Tumor multiplicity and malignancy grade reduced in DMBA model.	[70] (Brazil)
*Azadirachta indica* (Neem) leaf extract	In vivo (Two-stage CSC, DMBA/TPA)	Histo; PE; GE	Tumor burden reduced; tumor degeneration and reduced proliferation markers; NF-κB activity suppressed; STAT1 signaling enhanced.	[71] (India)
Oligonol	In vivo (Two-stage CSC, DMBA/TPA)	TInc/Mul; Prog/Surv TumP; TumPE	Papilloma incidence and multiplicity reduced; malignant conversion delayed; 100% survival at 40 weeks (high dose).	[72] (Korea)

Apop, apoptosis analysis; CC, cell-cycle analysis; CFA, colony formation assay; CIAG, clonogenic/anchorage-independent growth assay; CSC, chemical skin carcinogenesis; CVA, cell viability assay (colorimetric); DMBA, 7,12 dimethylbenz[a]anthracene; TPA, 12 O tetradecanoylphorbol 13 acetate; DNA dmg, DNA damage assessment; GE, gene expression analysis; Histo, histopathological analysis; IHC, immunohistochemistry; ICD, immune cell differentiation; MEA, mechanistic/target engagement assays; NR, not reported; MitF, mitochondrial function analysis; M, migration assay; M/I, migration/invasion assays; PDT, photodynamic therapy; Prolif, proliferation assay; RCD, regulated cell death; ROS, reactive oxygen species; TIC Prolif, tumor–immune co-culture proliferation assay; Tox, toxicity assessment; PE, protein expression analysis; WHA, wound healing assay; XG, xenograft model; Adh, cell adhesion assay; Surv, long-term survival assessment; Autoph, autophagy assays; NA-RCD, non-apoptotic regulated cell death assays; ρ^0^ cSCC cells, mitochondrial DNA-depleted (rho zero); Ferropt, ferroptotic cell death assessment; ImmDep, immunological/immune dependence assays; TumG, tumor growth assay; GSE, grape seed extract; RES, resveratrol; URA, ursolic acid; ELA, ellagic acid; TInc/Mul, tumor incidence and multiplicity assessment; Prog/Surv, tumor progression and survival analysis; TumPE, oncogenic enzyme expression analysis in tumors; UVB-SC, UVB-induced skin carcinogenesis; ex vivo (human IHC), immunohistochemical analysis of human tissue simples; TumP, tumor proliferation assay; TB/Inc/MalConv, tumor burden, incidence, and malignant conversion assessment; RNA seq, transcriptomic analysis of tumor and epidermal tissue.

**Table 2:** (**A**–**E**). Classification of natural products evaluated in cSCC models by preparation type, chemical composition, and mechanism of action. Compounds were first organized according to type of preparation. Preparations were then subclassified based on chemical class for purified compounds or prevalent (dominant) chemical class for chemically heterogeneous systems such as extracts, essential oils, and formulations. Mechanisms of action (MoAs) were grouped into the following categories using standardized abbreviations: APO, apoptosis; NA-RCD, non-apoptotic regulated cell death pathways; CCA, cell-cycle arrest-mediated growth suppression; EPI, epigenetic modulation of gene expression; SIG, inhibition of oncogenic signaling pathways; REDOX, oxidative stress- and redox-based mechanisms; PDT, photodynamic therapy-related cytotoxic mechanisms; CIM, chemopreventive and immune-mediated mechanisms; NSA, non-specific antiproliferative activity (antiproliferative effects without a dominant mechanistic assignment). (**A**) Purified compounds evaluated in cSCC models. (**B**) Natural extracts evaluated in cSCC models. (**C**) Essential oils evaluated in cSCC models. (**D**) Formulations and drug-delivery systems evaluated in cSCC models. (**E**) Combination treatments (non-formulated) evaluated in cSCC models.

**(A) Purified Compounds Evaluated In cSCC Models**								
**Preparation Type**	**Chemical Class**		**Subclass**	**Compound**	**Origin**		**MoA**	**Author**
Purified natural primary metabolites	Lipids		Short-chain fatty acid (SCFA)	Butyric acid (BA)	Gut microbiota fermentation of dietary fiber		APO	[69]
			Long-chain alkane	Tetracosane	*Helichrysum* *odoratissimum* (L.) Sweet		APO	[34]
	Carbohydrate		Carboxylated carbohydrate	Calcium glucarate (CAG)	Natural metabolite of glucose		APO	[69]
	Vitamin		Pyridine carboxamide	Nicotinamide (NA)	Present in food (meat, fish, grains, dairy)		APO	[69]
Isolated natural compounds (secondary metabolites)	Alkaloids		Indolo quinoline	Cryptolepine	*Cryptolepis* *sanguinolenta*		APO	[36]
			Indole alkaloids	Rescinnamine	*Rauwolfia* species		CCA	[20]
	Phenolic compounds—simple phenolics		Phenylpropanoid	2′- hydroxycinnamicadehyde (2′-HCA)	*Cinnamomum cassia*– cinnamon		APO	[37]
			Vanilloids (phenylpropanoid-derived phenolics)	[6]-Gingerol	*Zingiber officinale* (fresh ginger)		REDOX	[49]
				Capsaicin	*Capsicum* sp., *Euphorbia* *resinifera*		APO	[51]
				Resiniferatoxin	*Capsicum* sp., *Euphorbia* *resinifera*		APO	[51]
	Phenolic compounds—polyphenols		Binaphthyl polyphenols	Gossypol	Cottonseed		NA-RCD	[21]
			Diarylheptanoids	Curcumin	*Curcuma longa*		PDT, NA-RCD, SIG	[23,35,39]
			Flavonoids— catechins	Epigallocatechin gallate (EGCG)	*Camellia sinensis*		PTD, EPI	[23,24,48]
				(−)-Epigallocatechin (EGC)	*Camellia sinensis*		NSA	[48]
				(−)-Epicatechin- gallate (ECG)	*Camellia sinensis*		NSA	[48]
				(+)-Cyanidan-3-ol (CD-3)	*Acacia catechu* heartwood, mahogany, and black grape seeds		SIG	[56]
			Flavonoids—chalcone/ retrochalcone	Echinatin	Licorice (various *Glycyrrhiza* plants)		NA-RCD	[54]
				Licochalcone B (Lico B)	*Glycyrrhiza inflata* (chinese licorice)		APO	[33]
			Flavonoids— flavones	Apigenin	*Origanum vulgare*		APO	[44]
				Chrysin	*Hedychium spicatum/Propoleum*		NSA, CCA	[50,61]
				Nobiletin	Citrus fruits		REDOX	[57]
				Tectochrysin	*Hedychium spicatum*		NSA	[50]
			Flavonoids— isoflavones	Prunetin	*Dalbergia sissoo*		SIG	[22]
				Tectorigenin	*Dalbergia sissoo*		SIG	[22]
				Prunetin-4′-O-galactoside	*Dalbergia sissoo*		SIG	[22]
				Sissotrin	*Dalbergia sissoo*		SIG	[22]
				Biochanin-A	*Dalbergia sissoo*		SIG	[22]
			Flavonoids— flavonols	Quercetin	Various plants		PDT	[23]
				Fisetin	Fruits/vegetables		APO	[42]
			Naphthodianthrone	Hypericin (Hyp)	*Hypericum perforatum*		PDT	[46]
				Fagopyrin F	*Fagopyrum tataricum* (tartary buckwheat)		PDT	[18]
			Phenolic acid (dilactone)	Ellagic acid	Pomegranates, raspberries, strawberries, walnuts		CIM	[64]
			Stilbene	Resveratrol	Grape skins, red wine, peanuts, berries (blueberries, cranberries), *Polygonum cuspidatum*		CIM, SIG, NSA	[27,30,64]
				Pterostilbene	*Pterocarpus marsupium*, blueberries and grapes		CIM	[66]
			Xanthones	Alfa–mangostin	Mangosteen fruit (*Garcinia mangostana* L.)		SIG	[47]
				Mangiferin	*Mangifera indica* (mango tree)		REDOX	[52]
	Terpenoids		Diterpenoids	9-hydroxy- hedychenone	*Hedychium* *spicatum*		NSA	[50]
				Hedychilactone-B	*Hedychium* *spicatum*		NSA	[50]
				Hedychilactone-D	*Hedychium* *spicatum*		NSA	[50]
				Yunnacoronarin-A	*Hedychium* *spicatum*		NSA	[50]
				Hedychilactone C	*Hedychium* *spicatum*		NSA	[50]
			Triterpenoid	Bartogenic acid	*Barringtonia racemosa*		APO	[60]
				Ursolic acid	Apple peels, rosemary, basil, *Eriobotrya japonica*		APO, CIM	[38,64]
				Diosgenin	Fenugreek (*Trigonella foenum graecum*)		APO	[45]
				Glycyrrhizic acid	Licorice root (*Glycyrrhiza glabra*)		APO	[17]
			Tetraterpenoids	Lycopene	Tomatoes, watermelon, pink grapefruit		CIM	[64]
			Sesquiterpenoids	Ottelione	*Ottelia alismoides*		CCA	[20]
				Subamolide B	*Cinnamomum subavenium Miq.*		APO	[43]
			Monoterpenoid- derived compounds	Thymoquinone	*Nigella sativa* (black seed)		APO	[45,65]
**(B) Natural Extracts Evaluated In cSCC Models**								
**Preparation Type**	**Dominant Chemical Class**		**Origin**		**Solvent**		**MoA**	**Author**
Crude fungal extracts	Fungal secondary metabolites		*Aspergillus unguis*		Ethyl acetate		REDOX	[28]
			*Ganoderma tsugae*		Methanol		SIG	[63]
Crude plant extracts	Polyphenol- dominant extracts		*Vitis vinifera* (grapes seeds)		Ethanol		CIM	[64]
			*Punica granatum* (pomegranate fruit)		Methanol		APO	[65]
			*Persicaria capitata*		Ethanol		NSA	[13]
			*Helleborus purpurascens*		Ethanol		NSA	[26]
			Brazilian red propolis (resin from *Dalbergia ecastophyllum*)		Ethanol		NSA	[70]
			*Luisia tenuifolia*		Ethyl acetate		NSA	[19]
			*Nepenthes miranda*		Ethanol/methanol		APO	[15]
			*Polygonaceae* species (*Rumex acetosa*, *R. alpinus*, *R. aquaticus*, *R. scutatus*, *R. thyrsiflorus*)		Chloroform/n-hexane		NSA	[41]
	Polyphenol- dominant extracts (photosensitizer-enriched)		*Strobilanthes crispa*		Ethanol/ethyl acetate/n-hexane		PDT	[16]
			*Fagopyrum tataricum* (tartary buckwheat)		Ethanol		PDT	[18]
	Terpenoid- dominant extracts		*Hedychium spicatum*		Chloroform		NSA	[50]
			*Azadirachta indica*		Water		SIG	[71]
	Mixed-chemistry extract		*Curcuma longa* L.		Dichloromethane (DCM) extract		NA-RCD	[39]
			*Helichrysum odoratissimum*		Ethanol		APO	[34]
			*Dalbergia sissoo*		N-hexane		SIG	[22]
			*Luisia tenuifolia*		Ethanol		NSA	[19]
			*Ottelia alismoides*		Acetone		CCA	[20]
	Lipophilic extracts		*Luisia tenuifolia*		N-Hexane/chloroform		NSA	[19]
			*Nepenthes miranda*		Acetone		APO	[15]
	Saccharide- dominant extracts		*Uncaria tomentosa*		Water		APO	[31]
Semi-purified plant fractions	Polyphenol- dominant fractions		*Persicaria capitata*		N/A		NSA	[13]
			*Polygonaceae* family		N/A		NSA	[41]
	Polyphenol- dominant fractions (photosensitizer-enriched)		Fagopyrin F-containing fraction (FCF)		N/A		PDT	[18]
	Terpenoid- dominant fractions		*Helichrysum odoratissimum*		N/A		APO	[34]
	Peptide-dominant fractions		*Acanthus ebracteatus*		N/A		SIG	[29]
Crude plant preparations (non-solvent)	Mixed polyphenol –terpenoid-dominant		Fresh turmeric paste (*Curcuma longa*)		Water		APO	[68]
Crude protein extracts/hydrolysates	Protein/peptide mixture		*Acanthus ebracteatus*		Sodium dodecyl sulfate (SDS)		SIG	[29]
**(C) Essential Oils Evaluated In cSCC Models**								
**Dominant Chemical Class**	**Subclass**			**Source**			**MoA**	**Author**
Volatile terpenoid mixture	Monoterpene-dominant			*Mentha aquatica* L. cv. Lime			SIG	[53]
				*Mentha aquatica* var. Kenting			SIG	[58]
				Camphor white oil (*Cinnamomum camphora*)			CIM	[67]
				*Hedychium spicatum* seed			REDOX	[25]
	Sesquiterpene-dominant			*Pamburus missionis*			REDOX	[32]
**(D) Formulations And Drug-Delivery Systems Evaluated In Models**								
**Dominant Class**	**Compounds**		**Origin**	**Dominant** **Subclass**	**Formulation**		**MoA**	**Author**
Polyphenol- based	Oligonol		Grapeseed, lychee fruit	Proanthocyanidins, catechins	Standardized polyphenol mixture		CIM	[72]
	Quercetin		Various plants	Flavonoid	Nanoemulsion/nanoemulgel		NSA	[14]
	Curcumin C3 complex		*Curcuma longa*	Diarylheptanoid mixture	Standardized polyphenol mixture		SIG	[59]
	Curcumin C3 complex		*Curcuma longa*	Diarylheptanoid mixture	Lipid-vehicle oral formulation		SIG	[62]
Alkaloid- based	Lycorine		*Amaryllidaceae* spp.	Anisoquinoline alkaloid	Vesicular system (transfersomes)		APO	[55]
**(E) Combination Treatments (Non-Formulated) Evaluated In Cscc Models**								
**Combination** **Treatment**	**Compounds**			**Chemical Dominance**			**MoA**	**Author**
Isolated compound + crude extract	Thymoquinone + pomegranate extract			Mixed terpenoid–polyphenol			APO	[65]
Isolated compounds	Thymoquinone + diosgenin			Terpenoid-dominant			APO	[45]
Isolated compounds	Ursolic acid + resveratrol			Mixed polyphenol–terpenoid			NSA	[40]

## Data Availability

No new data were created or analyzed in this study. Data sharing is not applicable to this article.

## Supplementary Materials

The following supporting information can be downloaded at: https://www.mdpi.com/article/10.3390/ijms27125531/s1.

## Abbreviations

The following abbreviations are used in this manuscript:

cSCC	cutaneous squamous cell carcinoma
PRISMA	Preferred Reporting Items for Systematic Reviews and Meta-Analyses
ROS	reactive oxygen species
UV	ultraviolet
UVB	ultraviolet B
DNA	deoxyribonucleic acid
MAPK	mitogen-activated protein kinase
PI3K	phosphoinositide 3-kinase
Akt	protein kinase B
EGCG	epigallocatechin gallate
SCC	squamous cell carcinoma
PDT	photodynamic therapy
DMBA	7,12-dimethylbenz[a]anthracene
TPA	12-O-tetradecanoylphorbol-13-acetate
Apop	apoptosis analysis
CC	cell-cycle analysis
CFA	colony formation assay
CIAG	clonogenic/anchorage-independent growth assay
CSC	chemical skin carcinogenesis
CVA	cell viability assay (colorimetric)
DNA dmg	DNA damage assessment
GE	gene expression analysis
Histo	histopathological analysis
IHC	immunohistochemistry
ICD	immune cell differentiation
MEA	mechanistic/target engagement assays
MitF	mitochondrial function analysis
M	migration assay
M/I	migration/invasion assays
Prolif	proliferation assay
RCD	regulated cell death
TIC Prolif	tumor–immune co-culture proliferation assay
Tox	toxicity assessment
PE	protein expression analysis
WHA	wound healing assay
XG	xenograft model
Adh	cell adhesion assay
Surv	long-term survival assessment
Autoph	autophagy assays
NA-RCD	non-apoptotic regulated cell death assays
ρ^0^ cSCC cells	mitochondrial DNA-depleted (rho zero) cSCC cells
Ferropt	ferroptotic cell death assessment
ImmDep	immunological/immune dependence assays
TumG	tumor growth assay
GSE	grape seed extract
RES	resveratrol
URA	ursolic acid
ELA	ellagic acid
TInc/Mul	tumor incidence and multiplicity assessment
Prog/Surv	tumor progression and survival analysis
TumPE	oncogenic enzyme expression analysis in tumors
UVB-SC	UVB-induced skin carcinogenesis
ex vivo (human IHC)	immunohistochemical analysis of human tissue samples
TumP	tumor proliferation assay
TB/Inc/MalConv	tumor burden, incidence, and malignant conversion assessment
RNA seq	transcriptomic analysis of tumor and epidermal tissue
APO	apoptosis
CCA	cell-cycle arrest-mediated growth suppression
EPI	epigenetic modulation of gene expression
SIG	inhibition of oncogenic signaling pathways
REDOX	oxidative stress- and redox-based mechanisms
CIM	chemopreventive and immune-mediated mechanisms
NSA	non-specific antiproliferative activity
mTOR	mechanistic target of rapamycin
NF-κB	nuclear factor kappa B
AP-1	activator protein 1
STAT3	signal transducer and activator of transcription 3
EGFR	epidermal growth factor receptor
ERK	extracellular signal-regulated kinase
Wnt	wingless/integrated signaling pathway
β-catenin	beta catenin
Bax	Bcl-2-associated X protein
Bcl-2	B-cell lymphoma 2
VEGF	vascular endothelial growth factor
ATP	adenosine triphosphate
GSTM3	glutathione S-transferase Mu 3
PRDX2	peroxiredoxin 2
GPX4	glutathione peroxidase 4
xCT	cystine/glutamate antiporter (SLC7A11)
FTH1	ferritin heavy chain 1
RIP1	receptor-interacting protein kinase 1
RIP3	receptor-interacting protein kinase 3
DNMTs	DNA methyltransferases
HDACs	histone deacetylases
PP2A	protein phosphatase 2A
RelA (p65)	v-rel avian reticuloendotheliosis viral oncogene homolog A
HSP90α	heat shock protein 90 alpha
COX-2	cyclooxygenase 2
C/EBP	CCAAT/enhancer binding protein
MMP-2	matrix metalloproteinase 2
MMP-9	matrix metalloproteinase 9
MAL	methyl aminolevulinate
IC_50_	half-maximal inhibitory concentration
MTT	3-(4,5-dimethylthiazol-2-yl)-2,5-diphenyltetrazolium bromide
CCK-8	Cell Counting Kit-8
XTT	2,3-bis(2-methoxy-4-nitro-5-sulfophenyl)-2H-tetrazolium-5-carboxanilide
EGC	epigallocatechin
ECG	epicatechin gallate

## References

[1] Hedberg M.L., Berry C.T., Moshiri A.S., Xiang Y., Yeh C.J., Attilasoy C., Capell B.C., Seykora J.T. (2022). Molecular Mechanisms of Cutaneous Squamous Cell Carcinoma. Int. J. Mol. Sci..

[2] Jiang R., Fritz M., Que S.K.T. (2024). Cutaneous Squamous Cell Carcinoma: An Updated Review. Cancers.

[3] Winge M.C.G., Kellman L.N., Guo K., Tang J.Y., Swetter S.M., Aasi S.Z., Sarin K.Y., Chang A.L.S., Khavari P.A. (2023). Advances in cutaneous squamous cell carcinoma. Nat. Rev. Cancer.

[4] Cozma E.-C., Banciu L.M., Soare C., Cretoiu S.-M. (2023). Update on the Molecular Pathology of Cutaneous Squamous Cell Carcinoma. Int. J. Mol. Sci..

[5] Liu H.M., Cheng M.Y., Xun M.H., Zhao Z.W., Zhang Y., Tang W., Cheng J., Ni J., Wang W. (2023). Possible Mechanisms of Oxidative Stress-Induced Skin Cellular Senescence, Inflammation, and Cancer and the Therapeutic Potential of Plant Polyphenols. Int. J. Mol. Sci..

[6] Kowalski S., Karska J., Tota M., Skinderowicz K., Kulbacka J., Drąg-Zalesińska M. (2024). Natural Compounds in Non-Melanoma Skin Cancer: Prevention and Treatment. Molecules.

[7] Peterle L., Sanfilippo S., Borgia F., Li Pomi F., Vadalà R., Costa R., Cicero N., Gangemi S. (2023). The Role of Nutraceuticals and Functional Foods in Skin Cancer: Mechanisms and Therapeutic Potential. Foods.

[8] Aljabali A.A.A., Obeid M.A., Bashatwah R.M., Qnais E., Gammoh O., Alqudah A., Mishra V., Mishra Y., Khan M.A., Parvez S. (2025). Phytochemicals in Cancer Therapy: A Structured Review of Mechanisms, Challenges, and Progress in Personalized Treatment. Chem. Biodivers..

[9] Tian F., Sun S., Ge Z., Ge Y., Ge X., Shi Z., Qian X. (2025). Understanding the Anticancer Effects of Phytochemicals: From Molecular Docking to Anticarcinogenic Signaling. J. Nutr..

[10] Hidalgo L., Saldías-Fuentes C., Carrasco K., Halpern A.C., Mao J.J., Navarrete-Dechent C. (2022). Complementary and alternative therapies in skin cancer a literature review of biologically active compounds. Dermatol. Ther..

[11] Page M.J., McKenzie J.E., Bossuyt P.M., Boutron I., Hoffmann T.C., Mulrow C.D., Shamseer L., Tetzlaff J.M., Akl E.A., Brennan S.E. (2021). The PRISMA 2020 statement: An updated guideline for reporting systematic reviews. BMJ.

[12] Ndebia E.J., Kamsu G.T. (2025). Harnessing the Power of Natural Terpenoid Compounds Against Esophageal Squamous Cell Carcinoma: A Systematic Review. Future Pharmacol..

[13] Arya V., Gill A.K., Singh Y., Acharya A., Jamwal A. (2025). The Purified Fraction of Persicaria capitata Flowers Attenuates Proliferation in A-431 Cell Lines. Pharmacogn. Mag..

[14] Booravilli J., Sirisolla J.D. (2025). Assessment of Cytotoxic Effects of Quercetin Nanoemulgel on Different Skin Cancer cell lines. Drug Dev. Ind. Pharm..

[15] Lai K.M., Huang Y.H., Lien Y., Huang C.Y. (2025). Bioactive Potential of *Nepenthes miranda* Flower Extracts: Antidiabetic, Anti-Skin Aging, Cytotoxic, and Dihydroorotase-Inhibitory Activities. Plants.

[16] Chen C.S., Loke C.F., Poh T.V., Fong E.L., Lai O.M., Tan S.P. (2024). Phytochemicals Screening and Photocytotoxicity of *Strobilanthes crispa* (L.) Blume (Acanthaceae) Leaf Extracts. Malays. J. Chem..

[17] Mohamed D.D., Mahrous H., Khalil H., Ibrahim I.A., Mohamed D.D., Keshk O.S., Nada A.H., Maksoud A.I.A.E. (2024). Regulation of angiogenesis and inflammatory pathways by glycyrrhizic acid. Egypt. J. Dermatol. Venerol..

[18] Merin Rinky K., Gayathri Devi D., Priya V.K. (2024). Fagopyrin F fraction from Fagopyrum tataricum demonstrates photodynamic inactivation of skin infecting bacterium and squamous cell carcinoma (A431) cells. Photochem. Photobiol. Sci..

[19] Sethuraman S.P., Ramachandran K.P. (2024). Phytochemical Profiling, In-vitro Antioxidant and Cytotoxic Effects of *Luisia tenuifolia* Extracts Against Human Skin Squamous Carcinoma. Appl. Biochem. Biotechnol..

[20] Das S., Rahaman A., Nath R., Das Talukdar A., Nath D., Bhattacharjee S., Mandal D.P., Choudhury M.D., Das D., Das G. (2023). Effect of acetone fraction of *Ottelia alismoides* on the G2/M cell cycle arrest and apoptosis in the human carcinoma cell lines. J. Ethnopharmacol..

[21] Haasler L., von Montfort C., Kondadi A.K., Golombek M., Ebbert L., Wenzel C.K., Stahl W., Reichert A.S., Brenneisen P. (2023). Involvement of necroptosis in the selective toxicity of the natural compound (±) gossypol on squamous skin cancer cells in vitro. Arch. Toxicol..

[22] Naik H.N., Kanjariya D., Parveen S., Meena A., Ahmad I., Patel H., Meena R., Jauhari S. (2024). *Dalbergia sissoo* phytochemicals as EGFR inhibitors: An *in vitro* and *in silico* approach. J. Biomol. Struct. Dyn..

[23] Pivetta T.P., Vieira T., Silva J.C., Ribeiro P.A., Raposo M. (2023). Phototoxic Potential of Different DNA Intercalators for Skin Cancer Therapy: In Vitro Screening. Int. J. Mol. Sci..

[24] León D., Buchegger K., Silva R., Riquelme I., Viscarra T., Mora-Lagos B., Zanella L., Schafer F., Kurachi C., Roa J.C. (2020). Epigallocatechin Gallate Enhances MAL-PDT Cytotoxic Effect on PDT-Resistant Skin Cancer Squamous Cells. Int. J. Mol. Sci..

[25] Maurya A.K., Sharma A., Mukhia S., Rani D., Kumar A., Kumar D., Kumar R., Padwad Y.S., Chand G., Agnithotri V.K. (2022). Essential Oil Composition, In Vitro Biological Activities and Safety Evaluation of Cultivated Hedychium spicatum Seeds. Indian J. Pharm. Sci..

[26] Pilut C.N., Manea A., Macasoi I., Dobrescu A., Georgescu D., Buzatu R., Faur A., Dinu S., Chioran D., Pinzaru I. (2022). Comparative Evaluation of the Potential Antitumor of Helleborus purpurascens in Skin and Breast Cancer. Plants.

[27] Iqubal M.K., Chaudhuri A., Iqubal A., Saleem S., Gupta M.M., Ahuja A., Ali J., Baboota S. (2021). Targeted Delivery of Natural Bioactives and Lipid-nanocargos against Signaling Pathways Involved in Skin Cancer. Curr. Med. Chem..

[28] Kamat S., Kumari M., Taritla S., Jayabaskaran C. (2020). Endophytic Fungi of Marine Alga From Konkan Coast, India—A Rich Source of Bioactive Material. Front. Mar. Sci..

[29] Khamwut A., Jevapatarakul D., Reamtong O., T-Thienprasert N.P. (2019). *In vitro* evaluation of anti-epidermoid cancer activity of *Acanthus ebracteatus* protein hydrolysate and their effects on apoptosis and cellular proteins. Oncol. Lett..

[30] Zhang B., Lari Najafi M. (2020). Resveratrol inhibits skin squamous cell carcinoma proliferation, migration and invasion through up-regulating miR-126. Cell. Mol. Biol..

[31] Ciani F., Tafuri S., Troiano A., Cimmino A., Fioretto B.S., Guarino A.M., Pollice A., Vivo M., Evidente A., Carotenuto D. (2018). Anti-proliferative and pro-apoptotic effects of *Uncaria tomentosa* aqueous extract in squamous carcinoma cells. J. Ethnopharmacol..

[32] Pavithra P.S., Mehta A., Verma R.S. (2018). Induction of apoptosis by essential oil from *P. missionis* in skin epidermoid cancer cells. Phytomedicine.

[33] Kang T.H., Yoon G., Kang I.A., Oh H.N., Chae J.I., Shim J.H. (2017). Natural Compound Licochalcone B Induced Extrinsic and Intrinsic Apoptosis in Human Skin Melanoma (A375) and Squamous Cell Carcinoma (A431) Cells. Phytother. Res..

[34] Twilley D., Kishore N., Meyer D., Moodley I., Kumar V., Lall N. (2017). The effect of *Helichrysum odoratissimum* (L.) sweet on cancer cell proliferation and cytokine production. Int. J. Pharmacogn. Phytochem. Res..

[35] Jose A., Labala S., Venuganti V.V.K. (2017). Co-delivery of curcumin and STAT3 siRNA using deformable cationic liposomes to treat skin cancer. J. Drug Target..

[36] Pal H.C., Katiyar S.K. (2016). Cryptolepine, a Plant Alkaloid, Inhibits the Growth of Non-Melanoma Skin Cancer Cells through Inhibition of Topoisomerase and Induction of DNA Damage. Molecules.

[37] Kim J.E., Son J.E., Jeong H., Joon Kim D., Seo S.K., Lee E., Lim T.G., Kim J.R., Chen H., Bode A.M. (2015). A Novel Cinnamon-Related Natural Product with Pim-1 Inhibitory Activity Inhibits Leukemia and Skin Cancer. Cancer Res..

[38] Yang X., Li Y., Jiang W., Ou M., Chen Y., Xu Y., Wu Q., Zheng Q., Wu F., Wang L. (2015). Synthesis and Biological Evaluation of Novel Ursolic acid Derivatives as Potential Anticancer Prodrugs. Chem. Biol. Drug Des..

[39] Thongrakard V., Titone R., Follo C., Morani F., Suksamrarn A., Tencomnao T., Isidoro C. (2014). Turmeric toxicity in A431 epidermoid cancer cells associates with autophagy degradation of anti-apoptotic and anti-autophagic p53 mutant. Phytother. Res..

[40] Junco J.J., Mancha A., Malik G., Wei S.J., Kim D.J., Liang H., Slaga T.J. (2013). Resveratrol and P-glycoprotein inhibitors enhance the anti-skin cancer effects of ursolic acid. Mol. Cancer Res..

[41] Lajter I., Zupkó I., Molnár J., Jakab G., Balogh L., Vasas A., Hohmann J. (2013). Antiproliferative activity of polygonaceae species from the Carpathian Basin against human cancer cell lines. Phytother. Res..

[42] Pal H.C., Sharma S., Elmets C.A., Athar M., Afaq F. (2013). Fisetin inhibits growth, induces G_2_/M arrest and apoptosis of human epidermoid carcinoma A431 cells: Role of mitochondrial membrane potential disruption and consequent caspases activation. Exp. Dermatol..

[43] Yang S.Y., Wang H.M., Wu T.W., Chen Y.J., Shieh J.J., Lin J.H., Ho T.F., Luo R.J., Chen C.Y., Chang C.C. (2013). Subamolide B Isolated from Medicinal Plant Cinnamomum subavenium Induces Cytotoxicity in Human Cutaneous Squamous Cell Carcinoma Cells through Mitochondrial and CHOP-Dependent Cell Death Pathways. Evid. Based Complement. Altern. Med..

[44] Chan L.P., Chou T.H., Ding H.Y., Chen P.R., Chiang F.Y., Kuo P.L., Liang C.H. (2012). Apigenin induces apoptosis via tumor necrosis factor receptor- and Bcl-2-mediated pathway and enhances susceptibility of head and neck squamous cell carcinoma to 5-fluorouracil and cisplatin. Biochim. Biophys. Acta.

[45] Das S., Dey K.K., Dey G., Pal I., Majumder A., MaitiChoudhury S., Kundu S.C., Mandal M. (2012). Antineoplastic and apoptotic potential of traditional medicines thymoquinone and diosgenin in squamous cell carcinoma. PLoS ONE.

[46] Sharma K.V., Davids L.M. (2012). Hypericin-PDT-induced rapid necrotic death in human squamous cell carcinoma cultures after multiple treatment. Cell Biol. Int..

[47] Wang J.J., Sanderson B.J., Zhang W. (2012). Significant anti-invasive activities of α-mangostin from the mangosteen pericarp on two human skin cancer cell lines. Anticancer Res..

[48] Nandakumar V., Vaid M., Katiyar S.K. (2011). (-)-Epigallocatechin-3-gallate reactivates silenced tumor suppressor genes, Cip1/p21 and p16INK4a, by reducing DNA methylation and increasing histones acetylation in human skin cancer cells. Carcinogenesis.

[49] Nigam N., Bhui K., Prasad S., George J., Shukla Y. (2009). [6]-Gingerol induces reactive oxygen species regulated mitochondrial cell death pathway in human epidermoid carcinoma A431 cells. Chem. Biol. Interact..

[50] Reddy P.P., Rao R.R., Rekha K., Suresh Babu K., Shashidhar J., Shashikiran G., Vijaya Lakshmi V., Rao J.M. (2009). Two new cytotoxic diterpenes from the rhizomes of Hedychium spicatum. Bioorg. Med. Chem. Lett..

[51] Hail N., Lotan R. (2002). Examining the role of mitochondrial respiration in vanilloid-induced apoptosis. J. Natl. Cancer Inst..

[52] Jie L., Huang H., Sha L., Zhang L., Wang Y., Cao Y. (2026). Mangiferin targeting HSP90α induces DNA damage in cutaneous squamous cell carcinoma via the ROS/MAPK signaling pathway. Int. Immunopharmacol..

[53] Chang C.T., Chen Y.H., Shyur L.F. (2024). Phytocompounds from essential oil of Mentha aquatica L. cv. Lime prevent vemurafenib-promoted skin carcinogenesis via inhibiting HRAS(Q61L) keratinocytes and reprogramming macrophage activities. Phytomedicine.

[54] Kang Z., Wang P., Wang B., Yan Y., Zhao Z., Li C., Wen L., Wu M., Yan G., Wang X. (2024). Echinatin suppresses cutaneous squamous cell carcinoma by targeting GSTM3-mediated ferroptosis. Phytomedicine.

[55] Li Y., Tai Z., Ma J., Miao F., Xin R., Shen C., Shen M., Zhu Q., Chen Z. (2023). Lycorine transfersomes modified with cell-penetrating peptides for topical treatment of cutaneous squamous cell carcinoma. J. Nanobiotechnol..

[56] Monga J., Suthar S.K., Rohila D., Joseph A., Chauhan C.S., Sharma M. (2022). (+)-Cyanidan-3-ol inhibits epidermoid squamous cell carcinoma growth via inhibiting AKT/mTOR signaling through modulating CIP2A-PP2A axis. Phytomedicine.

[57] Bayoumi M., Arafa M.G., Nasr M., Sammour O.A. (2021). Nobiletin-loaded composite penetration enhancer vesicles restore the normal miRNA expression and the chief defence antioxidant levels in skin cancer. Sci. Rep..

[58] Chang C.T., Soo W.N., Chen Y.H., Shyur L.F. (2019). Essential Oil of Mentha aquatica var. Kenting Water Mint Suppresses Two-Stage Skin Carcinogenesis Accelerated by BRAF Inhibitor Vemurafenib. Molecules.

[59] Khandelwal A.R., Rong X., Moore-Medlin T., Ekshyyan O., Abreo F., Gu X., Nathan C.A. (2016). Photopreventive Effect and Mechanism of AZD4547 and Curcumin C3 Complex on UVB-Induced Epidermal Hyperplasia. Cancer Prev. Res..

[60] Patil C.R., Sonara B.M., Mahajan U.B., Patil K.R., Patil D.D., Jadhav R.B., Goyal S.N., Ojha S. (2016). Chemomodulatory Potential of Bartogenic Acid Against DMBA/Croton Oil Induced Two-Step Skin Carcinogenesis in Mice. J. Cancer.

[61] Liu H., Liu K., Huang Z., Park C.M., Thimmegowda N.R., Jang J.H., Ryoo I.J., He L., Kim S.O., Oi N. (2013). A chrysin derivative suppresses skin cancer growth by inhibiting cyclin-dependent kinases. J. Biol. Chem..

[62] Phillips J.M., Clark C., Herman-Ferdinandez L., Moore-Medlin T., Rong X., Gill J.R., Clifford J.L., Abreo F., Nathan C.O. (2011). Curcumin inhibits skin squamous cell carcinoma tumor growth in vivo. Otolaryngol. Head Neck Surg..

[63] Hsu S.C., Ou C.C., Chuang T.C., Li J.W., Lee Y.J., Wang V., Liu J.Y., Chen C.S., Lin S.C., Kao M.C. (2009). Ganoderma tsugae extract inhibits expression of epidermal growth factor receptor and angiogenesis in human epidermoid carcinoma cells: In vitro and in vivo. Cancer Lett..

[64] Kowalczyk M.C., Walaszek Z., Kowalczyk P., Kinjo T., Hanausek M., Slaga T.J. (2009). Differential effects of several phytochemicals and their derivatives on murine keratinocytes in vitro and in vivo: Implications for skin cancer prevention. Carcinogenesis.

[65] Allemailem K.S., Babiker A.Y., Khan A. (2025). Thymoquinone and Pomegranate Extract as Potential Chemopreventive Agents in a Murine Skin Carcinogenesis Model. Chem. Methodol..

[66] Surien O., Masre S.F., Basri D.F., Ghazali A.R. (2022). Chemopreventive Effects of Oral Pterostilbene in Multistage Carcinogenesis of Skin Squamous Cell Carcinoma Mouse Model Induced by DMBA/TPA. Biomedicines.

[67] Moayedi Y., Greenberg S.A., Jenkins B.A., Marshall K.L., Dimitrov L.V., Nelson A.M., Owens D.M., Lumpkin E.A. (2019). Camphor white oil induces tumor regression through cytotoxic T cell-dependent mechanisms. Mol. Carcinog..

[68] Rajmani R.S., Singh P., Singh L.V. (2017). Apoptotic and Immunosuppressive Effects of Turmeric Paste on 7, 12 Di Methyl Benz (a) Anthracene Induced Skin Tumor Model of Wistar Rat. Nutr. Cancer.

[69] Tiwari P., Sahay S., Pandey M., Qadri S.S., Gupta K.P. (2015). Combinatorial chemopreventive effect of butyric acid, nicotinamide and calcium glucarate against the 7,12-dimethylbenz(a)anthracene induced mouse skin tumorigenesis attained by enhancing the induction of intrinsic apoptotic events. Chem. Biol. Interact..

[70] Pinheiro K.S., Ribeiro D.R., Alves A.V., Pereira-Filho R.N., Oliveira C.R., Lima S.O., Reis F.P., Cardoso J.C., Albuquerque-Júnior R.L. (2014). Modulatory activity of Brazilian red propolis on chemically induced dermal carcinogenesis. Acta Cir. Bras..

[71] Arora N., Bansal M.P., Koul A. (2011). Azadirachta indica exerts chemopreventive action against murine skin cancer: Studies on histopathological, ultrastructural changes and modulation of NF-κB, AP-1,and STAT1. Oncol. Res..

[72] Kundu J.K., Hwang D.M., Lee J.C., Chang E.J., Shin Y.K., Fujii H., Sun B., Surh Y.J. (2009). Inhibitory effects of oligonol on phorbol ester-induced tumor promotion and COX-2 expression in mouse skin: NF-kappaB and C/EBP as potential targets. Cancer Lett..

[73] Chedid M.F., Tregnago A.C., Riva F., Prediger L., Agarwal A., Mattei J. (2025). Indications and Mechanisms of Action of the Main Treatment Modalities for Non-Melanoma Skin Cancer. Life.

[74] Bastos M., Saraiva L., Marques A.C., Amaral M.H. (2025). Skin cancer chemoprevention: An overview. Drug Discov. Today.

[75] Fulda S., Galluzzi L., Kroemer G. (2010). Targeting mitochondria for cancer therapy. Nat. Rev. Drug Discov..

[76] Galluzzi L., Vitale I., Aaronson S.A., Abrams J.M., Adam D., Agostinis P., Alnemri E.S., Altucci L., Amelio I., Andrews D.W. (2018). Molecular mechanisms of cell death: Recommendations of the Nomenclature Committee on Cell Death 2018. Cell Death Differ..

[77] Dixon S.J., Lemberg K.M., Lamprecht M.R., Skouta R., Zaitsev E.M., Gleason C.E., Patel D.N., Bauer A.J., Cantley A.M., Yang W.S. (2012). Ferroptosis: An iron-dependent form of nonapoptotic cell death. Cell.

[78] Rajendran P., Abdelsalam S.A., Renu K., Veeraraghavan V., Ben Ammar R., Ahmed E.A. (2022). Polyphenols as Potent Epigenetics Agents for Cancer. Int. J. Mol. Sci..

[79] Kroemer G., Galluzzi L., Vandenabeele P., Abrams J., Alnemri E.S., Baehrecke E.H., Blagosklonny M.V., El-Deiry W.S., Golstein P., Green D.R. (2009). Classification of cell death: Recommendations of the Nomenclature Committee on Cell Death 2009. Cell Death Differ..

[80] Indran I.R., Tufo G., Pervaiz S., Brenner C. (2011). Recent advances in apoptosis, mitochondria and drug resistance in cancer cells. Biochim. Biophys. Acta (BBA)—Bioenerg..

[81] Trachootham D., Alexandre J., Huang P. (2009). Targeting cancer cells by ROS-mediated mechanisms: A radical therapeutic approach?. Nat. Rev. Drug Discov..

[82] Reczek C.R., Chandel N.S. (2017). The Two Faces of Reactive Oxygen Species in Cancer. Annu. Rev. Cancer Biol..

[83] Williamson E.M. (2001). Synergy and other interactions in phytomedicines. Phytomedicine.

[84] Brendler T., Brinckmann J.A., Daoust M., He H., Masé G., Steffan K., Williams M. (2022). Suitability of botanical extracts as components of complex mixtures used in herbal tea infusions—Challenges and opportunities. Front. Pharmacol..

[85] Agostinis P., Berg K., Cengel K.A., Foster T.H., Girotti A.W., Gollnick S.O., Hahn S.M., Hamblin M.R., Juzeniene A., Kessel D. (2011). Photodynamic therapy of cancer: An update. CA Cancer J. Clin..

[86] Castano A.P., Demidova T.N., Hamblin M.R. (2005). Mechanisms in photodynamic therapy: Part two-cellular signaling, cell metabolism and modes of cell death. Photodiagnosis Photodyn. Ther..

[87] Dolmans D.E., Fukumura D., Jain R.K. (2003). Photodynamic therapy for cancer. Nat. Rev. Cancer.

[88] Allison R.R., Moghissi K. (2013). Photodynamic Therapy (PDT): PDT Mechanisms. Clin. Endosc..

[89] Sporn M.B., Suh N. (2000). Chemoprevention of cancer. Carcinogenesis.

[90] Schreiber R.D., Old L.J., Smyth M.J. (2011). Cancer immunoediting: Integrating immunity’s roles in cancer suppression and promotion. Science.

[91] Balkwill F., Mantovani A. (2001). Inflammation and cancer: Back to Virchow?. Lancet.

[92] Garraway L.A., Lander E.S. (2013). Lessons from the Cancer Genome. Cell.

[93] Hollingsworth S.J., Biankin A.V. (2015). The Challenges of Precision Oncology Drug Development and Implementation. Public Health Genom..

