# Supplementary material for: Natural Compounds for the Treatment of Cutaneous Squamous Cell Carcinoma: A Systematic Review
Source: Int J Mol Sci. 2026 Jun 18;27(12):5531. doi: 10.3390/ijms27125531 (PMC13299194; doi:10.3390/ijms27125531)
Supplement: Supplementary file 1 [file ijms-27-05531-s001.zip › Supplementary Table S1.pdf]

International Journal of *Molecular Sciences*

MDPI

Table S1. Supplementary Table S1. Key experimental parameters of *in vivo* studies included in this systematic review.

Natural Compounds/ Extract	Model	n/group	Dose*	Route	Duration	Frequency	Notes†	Ref.
Mangiferin	Xenograft	5	100 mg/kg	Oral	~4 weeks	q2d	Xenograft model; repeated dosing design.	[52]
Mentha aquatica L. cv. Lime oil	DMBA/TPA + PLX	NR	5 mg/site	Topical	12 weeks	2×/week	Combination chemical carcinogenesis model; sample size not reported.	[53]
Echinatin	Xenograft + UV	5–6	20–50 mg/kg	i.p.	~18 d	q3d	Dual model (xenograft + UV); multiple induction systems.	[54]
Lycorine	Xenograft	5	2.5 mg/kg	Topical	14 d	BID	Xenograft model; nanoparticle-based delivery system.	[55]
(+)-Cyanidan-3-ol	DMBA/TPA + xenograft	10	50–200 mg/kg	Oral	~20 weeks	3×/week	Dual model (chemical + xenograft); combined in vivo approaches.	[56]
Nobiletin	DMBA	15	NR	Topical	2 mo	Daily	Chemical carcinogenesis model; dose not reported.	[57]
Mentha aquatica oil	DMBA/TPA ± PLX	5–8	5 mg/site	Topical	12 weeks	2×/week	Chemical carcinogenesis model; combined treatment groups.	[58]
Curcumin	UVB	5	15 mg/kg	Oral	2 weeks	5×/week	UV-induced model; preventive treatment design.	[59]

Bartogenic acid	DMBA/croton oil	12	1–4 mg/kg	Oral + topical	~14 weeks	Daily / 2×/week	Chemical carcinogenesis model; combined oral and topical administration.	[60]
Chrysin	Xenograft	9	50 mg/kg	i.p.	3 weeks	q2d	Xenograft model; well-controlled study.	[61]
Curcumin	SCC xenograft	8	5–15 mg/day	Oral	24 d	Daily	Xenograft model; therapeutic design.	[62]
Ganoderma tsugae extract	Xenograft	6	~100 mg/kg	Oral	3 weeks	Daily	Xenograft model; dose derived from administration volume.	[63]
Proanthocyanidin extracts (GSE; RES; URA; ELA)	DMBA (short-term)	5	µmol range	Topical	4 weeks	2×/week	Chemical carcinogenesis model; short-term experimental design.	[64]
Thymoquinone + pomegranate extract	DMBA/TPA	5–10	20 mg/kg	Topical	4–22 weeks	3×/week	Chemical carcinogenesis model; multi-duration treatment protocol.	[65]
Pterostilbene	DMBA/TPA	6	50 mg/kg	Oral	24 weeks	~2×/week	Chemical carcinogenesis model; stage-specific design.	[66]
Camphor white oil	DMBA/TPA	10	2.5–40%	Topical	24 weeks	Daily	Chemical carcinogenesis model; stage-specific intervention design.	[67]
Fresh turmeric paste	DMBA	6–10	5% diet + topical	Oral + topical	28 d	Daily	Chemical carcinogenesis model; combined dietary and topical intervention.	[68]
Butyric acid, Nicotinamide,	DMBA	10	Multi-agent	Topical	4–16 weeks	2×/week	Chemical carcinogenesis model;	[69]

Calcium glucarate							multi-compound treatment design.	
Brazilian red propolis extract	DMBA	15	10–100 mg/kg	Oral	~6 weeks	q2d	Chemical carcinogenesis model; short-duration protocol.	[70]
Azadirachta indica (Neem) leaf extract	DMBA/TPA	6–8	300 mg/kg	Oral	20 weeks	3×/week	Chemical carcinogenesis model; lack of reported blinding.	[71]
Oligonol	DMBA/TPA	25	1–10 mg	Topical	20–40 weeks	2×/week	Chemical carcinogenesis model; long-term experimental protocol.	[72]

Ref., reference; DMBA, 7,12-dimethylbenz[a]anthracene; TPA, 12-O-tetradecanoylphorbol-13-acetate; PLX, PLX4032 (vemurafenib); SCC, squamous cell carcinoma; NR, not reported; i.p., intraperitoneal; BID, twice daily; q2d, every two days; q3d, every three days; d, days; mo, months; UV, ultraviolet radiation.

*Dose units are reported as described in the original studies.

†Notes summarize objective experimental features of each study, including model structure, treatment design, and reporting characteristics, without formal quality grading.
